# Inhibition of Thrombopoietin/Mpl Signaling in Adult Hematopoiesis Identifies New Candidates for Hematopoietic Stem Cell Maintenance

**DOI:** 10.1371/journal.pone.0131866

**Published:** 2015-07-06

**Authors:** Saskia Kohlscheen, Sabine Wintterle, Adrian Schwarzer, Christel Kamp, Martijn H. Brugman, Daniel C. Breuer, Guntram Büsche, Christopher Baum, Ute Modlich

**Affiliations:** 1 Research Group for Gene Modification in Stem Cells, LOEWE Center for Cell and Gene Therapy Frankfurt/Main and the Paul-Ehrlich-Institute, Langen, Germany; 2 Institute of Experimental Hematology; Hannover Medical School, Hannover, Germany; 3 Department of Biostatistik, Paul-Ehrlich-Institute, Langen, Germany; 4 Department of Immunohematology and Blood Transfusion, Leiden University Medical Center, ZA Leiden, The Netherlands; 5 Institute of Pathology, Hannover Medical School, Hannover, Germany; French Blood Institute, FRANCE

## Abstract

Thrombopoietin (Thpo) signals via its receptor Mpl and regulates megakaryopoiesis, hematopoietic stem cell (HSC) maintenance and post-transplant expansion. Mpl expression is tightly controlled and deregulation of Thpo/Mpl-signaling is linked to hematological disorders. Here, we constructed an intracellular-truncated, signaling-deficient Mpl protein which is presented on the cell surface (dnMpl). The transplantation of bone marrow cells retrovirally transduced to express dnMpl into wildtype mice induced thrombocytopenia, and a progressive loss of HSC. The aplastic BM allowed the engraftment of a second BM transplant without further conditioning. Functional analysis of the truncated Mpl *in vitro* and *in vivo* demonstrated no internalization after Thpo binding and the inhibition of Thpo/Mpl-signaling in wildtype cells due to dominant-negative (dn) effects by receptor competition with wildtype Mpl for Thpo binding. Intracellular inhibition of Mpl could be excluded as the major mechanism by the use of a constitutive-dimerized dnMpl. To further elucidate the molecular changes induced by Thpo/Mpl-inhibition on the HSC-enriched cell population in the BM, we performed gene expression analysis of Lin-Sca1+cKit+ (LSK) cells isolated from mice transplanted with dnMpl transduced BM cells. The gene expression profile supported the exhaustion of HSC due to increased cell cycle progression and identified new and known downstream effectors of Thpo/Mpl-signaling in HSC (namely TIE2, ESAM1 and EPCR detected on the HSC-enriched LSK cell population). We further compared gene expression profiles in LSK cells of dnMpl mice with human CD34+ cells of aplastic anemia patients and identified similar deregulations of important stemness genes in both cell populations. In summary, we established a novel way of Thpo/Mpl inhibition in the adult mouse and performed in depth analysis of the phenotype including gene expression profiling.

## Introduction

The hematopoietic cytokine thrombopoietin (Thpo) signals through its receptor Mpl, which is expressed on megakaryocytes/platelets and hematopoietic stem cells (HSC), and mediates megakaryopoiesis and HSC maintenance [1]. Thpo binding induces dimerization of Mpl receptors, causing activation of bound Janus kinases (JAK2 and TYK2) and subsequent phosphorylation of tyrosine residues of the intracellular domains of the Mpl receptor. Downstream signaling pathways activate STAT3/5, PI3K/AKT and MAPK/ERK. Constitutive MPL activation is found in myeloproliferative neoplasms underlining the importance for controlled MPL-signaling [2,3].

Absence of Mpl-signaling in *Mpl-/-* and *Thpo-/-* mice causes thrombocytopenia and HSC defects [4,5]. In competitive repopulation assays, *Mpl-/-* bone marrow (BM) cells repopulated wildtype (wt) recipient mice less potently than wt cells [6,7]. Furthermore, Mpl-signaling is essential for post-transplant expansion of HSC [8]. MPL deficiency in humans causes the rare inherited disease congenital amegakaryocytic thrombocytopenia (CAMT), which first presents with thrombocytopenia and develops to aplastic anemia [9,10]. Otherwise lethal, CAMT is currently treated with allogeneic HSC transplantation early in childhood [11].

In our previous work, we developed gene therapy approaches to treat *MPL* deficiency using wt and *Mpl-/-* mouse models [12,13]. We demonstrated the correction of the thrombocytopenia and stem cell defects by lentiviral Mpl expression and transplantation of the transduced BM into *Mpl*-/- mice. However, Mpl expression had to be tightly restricted to its physiological sites of expression, namely megakaryocytes, platelets and HSC. The ectopic expression of Mpl in wt mice induced thrombocytopenia and pancytopenia [12], similar to the earlier reports by Yan 1999 [14]. These thrombocytopenic mice had low Thpo levels and could be rescued by the application of Thpo, indicating a competition of the ectopic and endogenous Mpl for Thpo binding and subsequent internalization. Also the number of LSK cells was reduced in these mice, therefore peripheral Thpo levels may also control the HSC compartment. In line with this observation, in a study by de Graaf and colleagues HSC defects were induced by Thpo-independent thrombocytosis in *Myb* ^Plt4/Plt4^ mice [15,16]. Thpo is constantly produced by the liver [17] and to some extent by BM stroma cells [18] and Thpo levels are controlled by receptor-mediated internalization and subsequent degradation [19]. In *Myb* ^Plt4/Plt4^ mice, overall Mpl was increased due to increased platelet numbers and therefore decreasing the Thpo levels.

However, in all these experiments, the full length Mpl was expressed. Therefore it cannot be excluded that alterations in Mpl signaling by ectopic expression of Mpl in HSC or MK was also involved in the phenotype. To address this question we developed an Mpl receptor that would bind Thpo but not transmit any signals by the truncation of the intracellular Mpl domains. We hypothesized that the ectopic expression of Thpo-binding sites in the peripheral blood will interfere with the Thpo-Mpl balance and as a consequence alter HSC behavior also when wtMpl in HSC was present. In our experiments, the truncated Mpl receptor was retrovirally overexpressed in a BM transplantation model in wt mice and the consequence in hematopoiesis and the stem cell compartment was investigated. To further elucidate the molecular changes in the HSC-enriched LSK cell population after inhibition of Thpo-Mpl interaction we performed gene expression analysis and identified new and known Mpl targets.

## Materials and Methods

### Animals

Animal experiments were approved by the local ethical committee (Lower Saxony State Office for consumer protection and Food Safety) and performed according to their guidelines. During the first three weeks after lethal irradiation and transplantation the mice were monitored once or twice per day for their state of health. To circumvent further complications or suffering of the mice by infections after the irradiation antibiotics (Ciprofloxacin 0.1mg/ml) were supplied to the mice by the drinking water. Afterwards, the mice were monitored every 2–3 days for their health according to the defined humane endpoints. In case mice were found to be ill (hunched posture, limited mobility, ruffled fur, anemic) or at the end of the observation time of the experiment, mice were euthanized with CO2 and subsequent cervical dislocated. C57BL/6 [B6.Ly5.2] and C57BL/6 PeP3b [B6 SJL/Ly5.1] mice were obtained from Janvier. *Mpl-/-* mice were kindly provided by W. Alexander, WEHI Institute Australia [20]. All mice were bred and kept in the specified pathogen-free animal facilities of the Hannover Medical School, Germany.

### Gammaretroviral vectors and vector production

The gammaretroviral vector RSF91 (kindly provided by Axel Schambach, Hannover Medical School, [21]) was used for the expression of the truncated, dominant-negative (dn)Mpl, the truncated and dimerized (cd-dn)Mpl, wildtype (wt)Mpl, truncated human CD34 (trCD34, retained 16 aa of the intracellular domain [22]) and GFP. The RSF91 vector is a conventional gammaretroviral vector which expresses from the long terminal repeats (LTRs) containing the spleen focus forming enhancer/promoter. For detection of the wildtype and truncated Mpl, the hemagglutinine tag (HA-tag) was added at bp 78 between the signal peptide and the extracellular domain. In all except experiment 2, vectors co-expressed GFP by an internal ribosomal entry site (IRES) for the detection of transduced cells were used. For *in vitro* studies a self inactivating gammaretroviral vector (SRS11) expressing the HA-wtMpl from the phosphoglycerate kinase promotor (PGK) was applied [23]. Vectors were produced in 293T cells by co-transfection of the transgene expressing vector with viral-gag/pol (pcDNA3.MLVg/p) and viral-env (K73eco) using the calcium phosphate transfection method. Viral vector titres were estimated by transduction of murine fibroblasts (SC1 cells).

### BM cell purification, transduction and transplantation

BM cells were flushed from the femurs and tibias of C57Bl/6 donor mice. Lineage-marker negative (lin-) cells were isolated by magnetic cell sorting using lineage-specific antibodies (GR1, CD11b, CD45R/B220, CD3e, TER-119; Milteny Biotech, Bergisch Gladbach, Germany). Prior to viral transduction, lin- BM cells were prestimulated for 24-48h in StemSpan (CellSystems, St. Katharinen, Germany), containing 10 ng/ml murine SCF, 20 ng/ml murine Thpo, 10 ng/ml recombinant human FGF-1, 20 ng/ml murine IGF2, 1% penicillin/streptomycin, 2 mM glutamine. Lin- cells were transduced twice on two following days with an MOI of 10 with retroviral vectors on Retronectin coated and preloaded wells (10 μg/cm^2^) [12]. On day four after isolation, 5x10^5^ cells/mouse were intravenously injected into lethally irradiated C57Bl/6 mice (10 Gy).

### Mouse analysis and flow cytometry

Peripheral blood was collected by retro-orbital bleeding and analyzed by automated blood cell counts (Scil ABC Vet Blood Counter, ABX Diagnostics, France).

At the end of the observation time, cells from BM, spleen and blood were subjected to flow cytometry (Becton Dickinson, Heidelberg, Germany). Antibodies were usually directly linked to FITC, PE, APC, Alexa488, Alexa700, PerCP-Cy5.5, or PE-Cy7 fluorochromes (Becton Dickinson, France, eBioscience, California, USA or Roche diagnostics, Mannheim, Germany). Dead cells were excluded by propidium iodide (PI) or 4',6-Diamidino-2-phenylindoledihydrochloride (DAPI) staining. For determination of cell cycle status, cells were stained first for extracellular markers and fixed in 2% PFA for 10 min. Subsequently cells were stained with anti-Ki67-PE for 1h in a 0.5% saponin buffer, and Hoechst 33342 was added for 15min.

For analysis of signal transduction, cells were fixed in 2% PFA and permeabilized with methanol. Subsequently, intracellular staining for phosphorylated STAT5, and ERK1/2 with Alexa Fluor 647 coupled antibodies (BDbiosciences, Heidelberg, Germany) was performed.

### Histo-Pathology

Organs of mice were fixed in 4% formalin for at least 24h and embedded in paraffin. In the case of the bones, they were decalcified by ethylene-diamine tetra-acetic acid. 3 μm sections were cut and stained with Hematoxylin/Eosin.

### Thpo quantification

Plasma was separated by centrifugation of whole blood at 400xg for 30-45min and stored at -20°C. Quantification of Thpo in mouse plasma was performed using a Thpo ELISA (R&D Systems; Minneapolis, MN; USA).

### Western Blot and EMSA

Transfected 32D cells were starved overnight in minimal media (RPMI, 4% BSA) and stimulated on the following day with cytokines for 10 min (20 ng/ml Thpo, 5 ng/ml IL3, PeproTech GmbH, Hamburg, Germany). Cells were then washed in ice cold PBS with phosphatase inhibitors, pelleted, and lysed in 50mM HEPES, 150mM NaCl, 50mM NaF, 10 mM Na_4_P_2_O_7_, 10% Glycerin, 1% Triton X-100 including phosphatase and protease inhibitors and frozen to -80°C. 10–20 μg of protein samples were separated by electrophoresis on a 9% acryl amid gel as described [24] Nitrocellulose membrane was blocked in 5% milk and incubated with anti-pERK (p44/42, T202/Y204) and anti-pAKT (Ser473) antibodies or anti-ERK and anti-AKT antibodies as loading controls (Cell Signaling, Technologies, United States). Same protein lysates as for Western blots were also used for Electromobility Shift Assays (EMSA). In the EMSA, DNA substrates beta-casein for STAT5 and USTE-oligo for STAT3 were p32-γ-ATP radioactively labeled and incubated (30 min. at room temperature) with the protein lysates (1–2 μg). Afterwards, DNA/protein mixtures were separated by electrophoresis (20% acryl amid gel, 2-3h, 300 volt) and analyzed by autoradiography [25].

### Microarray analysis

LSK cells were isolated from dnMpl and control transplanted mice by fluorescent activated cell sorting. For each sample, BM of 2–5 mice were pooled. RNA was isolated using RNeasy Micro Kit (Qiagen GmbH, Hilden, Germany). RNA quality was assessed using the Agilent 2100 Bioanalyzer. RNA was reverse transcribed, amplified and labelled using the Nugen Ovation Pico and Encore biotin kits (Nugen Technologies, AC Bemmel, Netherlands). The resulting material was hybridized to Affymetrix Mouse 430 2.0 arrays. Data were analyzed using R and Bioconductor [26]. Array quality was checked with the ArrayQualityMetrics package [27]. Arrays were background corrected, normalized and summarized using RMA [28]. We used LIMMA to detect differentially expressed probe sets applying the Benjamini-Hochberg procedure for correction of multiple testing. For testing enrichment of gene sets the Broad Institute GSEA software package was employed [29]. The datasets were collapsed into single genes and rank-ordered by signal to noise ratio. 1000 Gene set permutations were used to estimate statistical significance. Analyzed gene sets were obtained from MSigDB and GeneSigDB [29,30]. The comparison of gene expression profiles in dnMpl LSK cells in CD34+ cells of patients with severe aplastic anemia (SAA) and refractory cytopenia (RC) was made on the basis of the signal to noise ratio score given in the rank ordered gene lists derived with the GSEA software package.

### Statistical analysis

For comparison of two experimental groups, we used the two-tailed unpaired t-test with Welch’s correction.

## Results

### The intracellular-truncated Mpl cannot transmit Thpo induced signals

**Fig 1:**
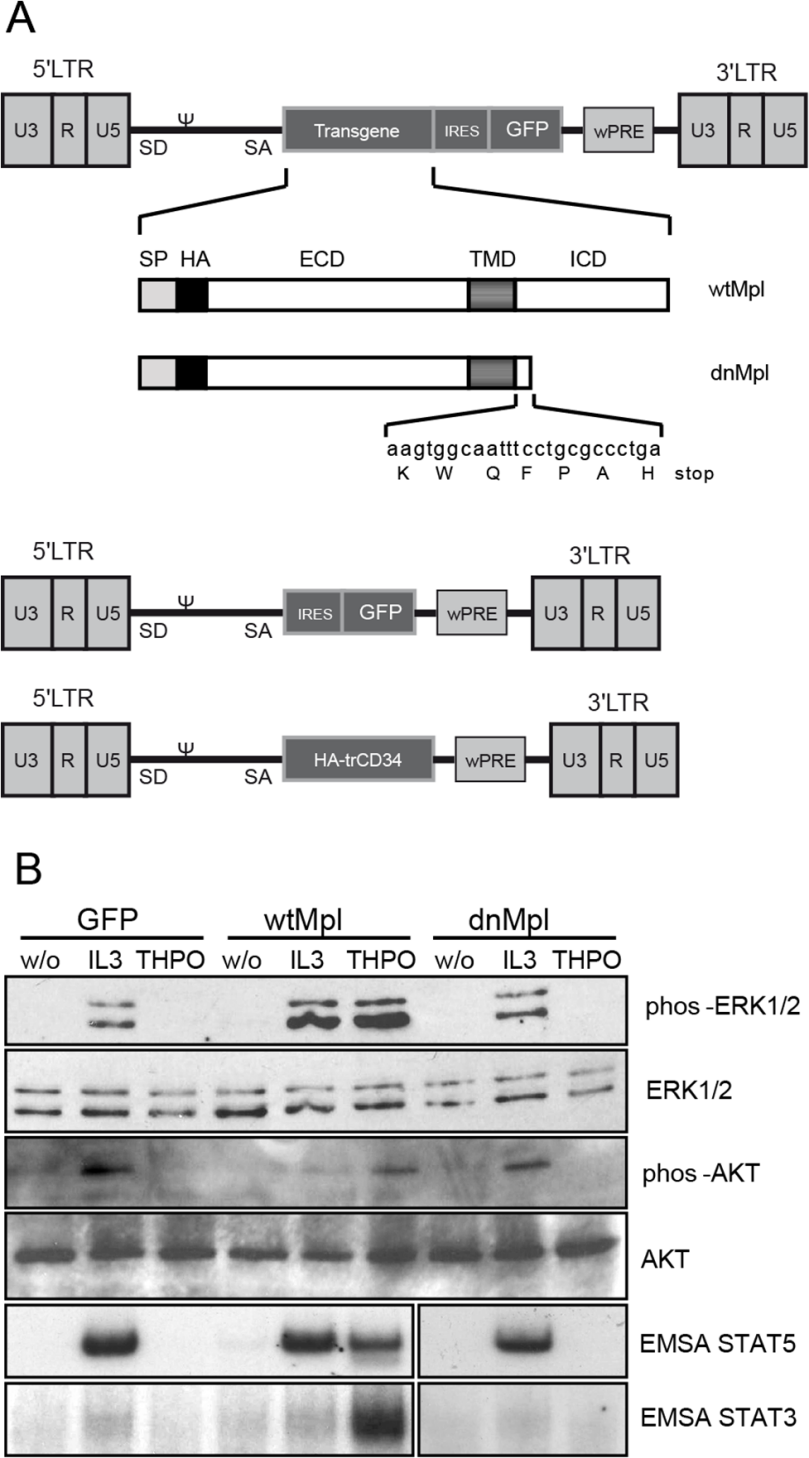
dnMpl does not transmit Thpo induced signals. (A) The gammaretroviral LTR vector encoded the full length or the intracellular truncated, dominant-negative (dn)Mpl cDNA. For detection of the Mpl proteins a hemagglutinin (HA)Tag was added at the N-terminus between the signal peptide and the ECD. The vector also co-expressed GFP using an internal ribosomal entry site (IRES). As control the retroviral vector only containing IRES.GFP or a truncated form of human CD34 was used. (LTR: long terminal repeat, ψ: packaging signal, SD: splice donor, SA: splice acceptor, wPRE: Woodchuck hepatitis virus posttranscriptional regulatory element, SP: signal peptide, ECD: extracellular domain, TMD: transmembrane domain, ICD: intracellular domain). (B) Western blot analysis of Mpl downstream signaling proteins in 32D cells that were transduced with wtMpl, dnMpl or GFP as a control. Transduced cells were stimulated with mThpo (20ng/mL), IL-3 (5ng/ml) or fixed without stimulation. Activation of STAT3 and STAT5 was analyzed by EMSA. No phosphorylation of ERK1/2, AKT and STATS was detected in dnMpl expressing 32D cells after Thpo stimulation similar to the GFP control transduced cells.

We constructed gammaretroviral vectors encoding a truncated Mpl receptor lacking the intracellular domain leaving only seven amino acids C-terminal of the transmembrane domain (termed dominant-negative (dn)Mpl, Fig 1A). As neutral controls, retroviral vectors were generated that expressed GFP or the human truncated (tr)CD34 as marker proteins and as positive control for *in vitro* experiments we expressed the wildtype (wt)Mpl (Fig 1A). For detection of the transgenic Mpl or truncated CD34, an HA tag was added at the extracellular N-terminus between the signal peptide and the ECD. HA-trCD34 was used in experiment 2 to allow equal staining procedures to the HA-Mpl on the target cell populations. To test the functionality of the expressed Mpl, 32D cells were transduced with the respective vectors and stimulated with Thpo. 32D cells transduced with dnMpl did not transmit Thpo mediated signals, while Thpo stimulation of wtMpl-expressing 32D cells induced ERK, AKT, STAT3 and STAT5 phosphorylation at similar or higher levels than after stimulation with IL3 (Fig 1B) indicating that Thpo binding to the transgenic receptor was possible. In addition, transduced 32D cells were stained for the presence of the HA-tagged protein on the cell surface showing equal protein expression (S1A Fig).

We then investigated the effects of dnMpl expression on hematopoiesis *in vivo* in a BM transplantation (BMT) model in C57BL/6 wt mice. Lineage marker negative (Lin-) BM donor cells were transduced *in vitro* with the gammaretroviral vector expressing dnMpl or, as controls, GFP or trCD34. Transduction rates varied between 30% and 80% *in vitro* before transplantation. We performed three independent experiments (experiment 1–3, Table 1), and two further experiments (experiment 4 and 5) to address further questions during our study.

**Table 1:** Overview of performed mouse experiments.

Experi-ment	Transgene	Number of mice	% transgene positive cells (PB, 5–8 weeks post Tx)	Observation time (weeks)	Final diagnosis
**1**	dnMpl-IRES.GFP	5	2–22	29	thrombocytopenic
**1**	IRES.GFP*	5	27–38	29	healthy
**2**	dnMpl	10	21–53	16	thrombocytopenic
**2**	trCD34	10	33–71	16	healthy
**3**	dnMpl- IRES.GFP	5	65–75	23**	thrombocytopenic
**3**	cd-dnMpl- IRES.GFP	5	66–75	23**	thrombocytopenic
**3**	IRES.GFP*	5	78–83	23**	healthy
**4**	dnMpl- IRES.GFP	20	56–73	8	Exp. terminated to collect LSK cells
**4**	IRES.GFP*	10	85–90	8	Exp. terminated to collect LSK cells
**5**	dnMpl- IRES.GFP	4	1–14	19+20***	Sec. BM transplant into same recipient
5	IRES.GFP*	4	7–25	19+20***	Sec. BM transplant into same recipient

*IRES.GFP mice of the control group are named GFP throughout the manuscript.

** Two mice of each group were transplanted with a second BM graft (2 x 10^7^ cells) 19 weeks after the first transplantation and followed for additional 16 weeks.

*** All mice were transplanted with a second BM graft (2 x 10^7^ cells) 19 weeks after the first transplantation and followed for additional 20 weeks

### Retroviral expression of dnMpl in wildtype mice causes thrombocytopenia

**Fig 2:**
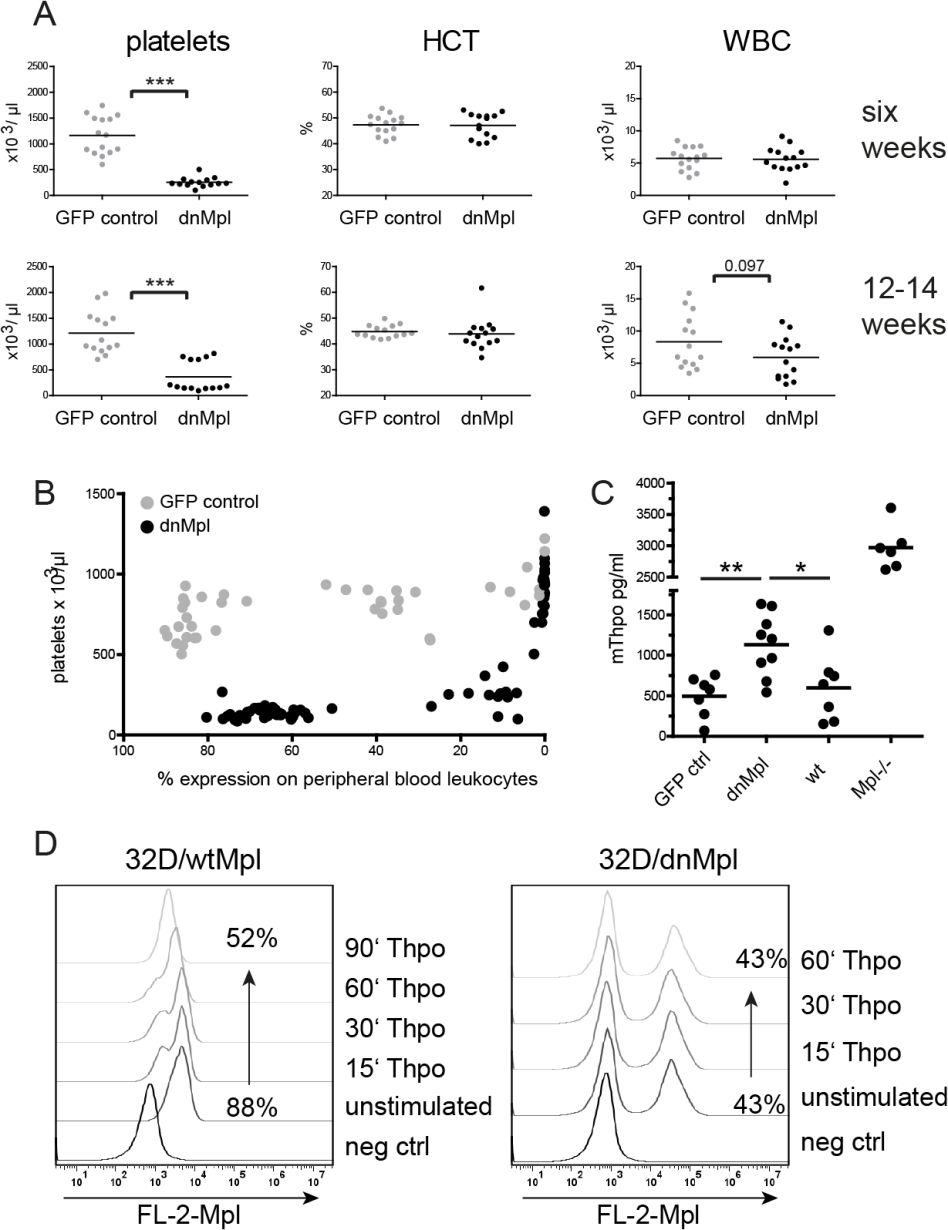
dnMpl expression *in vivo* causes thrombocytopenia. (A) Blood cell counts of transplanted mice six and 12–14 weeks after transplantation. Platelet counts remained significantly reduced in the dnMpl group, whereas, the hematocrit was unchanged in both groups. Slightly reduced WBC counts were observed at late time points after transplantation in the dnMpl group. (*** p<0.005, Students t-test) HCT: hematocrit, WBC: white blood cells. (B) Correlation of dnMpl expression on leukocytes with platelet counts. Blood of mice transplanted with dnMpl or GFP control transduced BM cells was taken at different time points after transplantation, platelet counts and transgene expression were measured by automated blood cell counts or flow cytometry, respectively. dnMpl expression results in decreased platelet counts. With declining dnMpl expression the platelet counts adjust to physiological levels. (C) Thpo plasma levels were determined in the blood of transplanted mice by ELISA, 12 weeks after transplantation or untransplanted wildtype and *Mpl-/-* mice. (*p<0.05, **p<0.01, Students t-test). (D) Receptor uptake in 32D cells. 32D cells were transduced with either wtMpl or dnMpl expressing vectors and stimulated with 50ng/mL mThpo or not. Cells were then fixed at different time points after Thpo has been added and surface expression of either receptor was measured by flow cytometry using an anti-HA Biotin conjugated primary and Streptavidin-PE secondary antibody.

In the experiments 1 and 2, we transplanted five and ten wt mice per group with BM cells expressing dnMpl, GFP or trCD34 as control (Table 1). Cell surface expression of dnMpl and trCD34 on peripheral blood leukocytes was confirmed by live cell staining and analysis of the HA-tag using flow cytometry (S1B Fig). dnMpl expression was detected on all hematopoietic lineages in the peripheral blood of transplanted mice by flow cytometric analysis (S2 Fig). Platelet counts were severely reduced in dnMpl mice six weeks after BMT (254+/-25 versus 1162+/-95 x 10^3^/μl in control mice, p<0.0001) and remained low (Fig 2A) with the exception of five mice with low levels of dnMpl expression. We found that ~6% of dnMpl positive cells in the blood (based on the expression in leukocytes) were sufficient to induce thrombocytopenia (Fig 2B). The hematocrit was unaffected by the expression of dnMpl, but 12 to 14 weeks after BMT, dnMpl mice showed a tendency of reduced white blood cell (WBC) counts (5.9+/- 0.86 x10^3^/μl versus 8.34+/-1.11 x10^3^/μl in control mice, p = 0.097). However, the expression of dnMpl did not affect the hematopoietic contribution of the different leukocyte lineages in the blood (S3 Fig). Thus, dnMpl expression had no effect on hematopoietic differentiation of non-thrombocytic lineages.

As Thpo is internalized and degraded after binding to Mpl, blood Thpo levels are controlled by platelet numbers [31], however, low Thpo can also be causative for thrombocytopenias. Therefore, we analyzed Thpo concentrations in the plasma of dnMpl mice. Thpo levels were elevated (1131 ± 128.7 pg/ml plasma, n = 9) 12 weeks after transplantation (Fig 2C). High Thpo levels were also found in Mpl deficient mice where Thpo is not cleared by receptor binding and internalization. The dnMpl receptor lacked most of the intracellular domains essential for its internalization and lysosomal targeting [19]. Therefore, we hypothesized, that Thpo binding to dnMpl did also not lead to internalization and degradation in our model. To test this 32D cells were transduced with either wtMpl or dnMpl vectors and analyzed for receptor uptake by flow cytometry. While we observed wtMpl to be cleared from the cell surface, dnMpl surface expression did not change even after one hour exposure to high Thpo concentrations (Fig 2D) confirming that dnMpl was not internalized after Thpo binding. However, the elevated Thpo levels in the blood of dnMpl mice did not increase platelet production although the endogenous wtMpl receptor was present. On the contrary, 12–14 weeks after transplantation also the leukocyte counts dropped, indicating insufficient blood cell production in the BM.

### dnMpl competes for Thpo binding with the wildtype Mpl receptor

**Fig 3:**
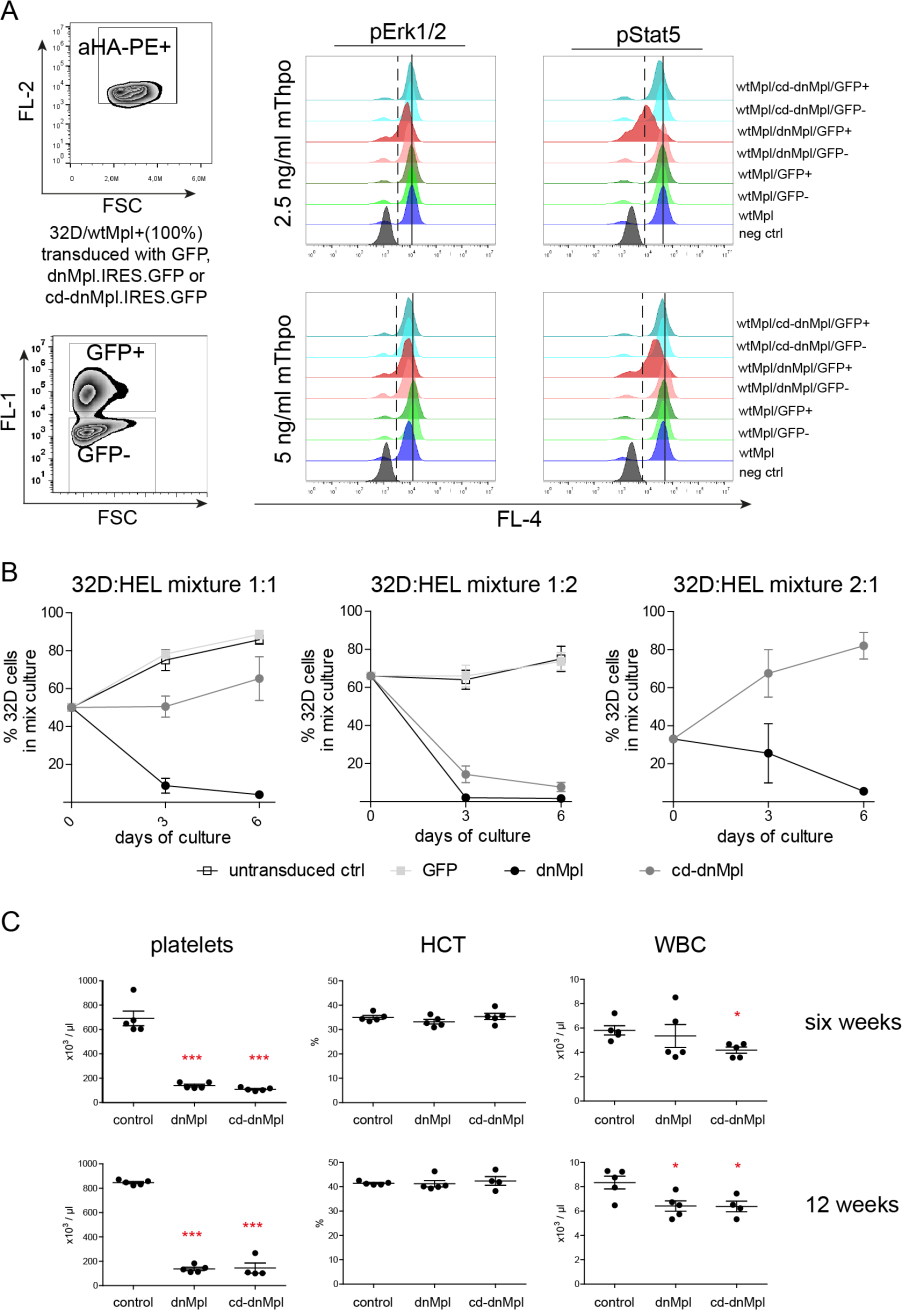
Systemic and cell intrinsic effects of dnMpl expression. (A) wtMpl expressing 32D cells (aHA-PE positive) were transduced with dnMpl.IRES.GFP, constitutive dimerized (cd)-dnMpl.IRES.GFP or GFP encoding vectors to establish cultures with single and double positive cells. Cells were starved for any cytokine stimuli for 16 hrs and stimulated with 2.5 or 5 ng/mL mThpo for 15 minutes the next day. Unstimulated (negative control) and stimulated cells were fixed and permeabelized to allow intracellular staining of phosphorylated signaling molecules. Anti-phosphoERK1/2 or phosphoSTAT5 antibodies conjugated to Alexa Fluor 647 (BD Biosciences) were used. Shown are histogram overlays of pERK1/2 and pSTAT5 activation from wtMpl/GFP negative cells and wtMpl/GFP, wtMpl/dnMpl, wtMpl/cd-dnMpl double positive cells. (Dashed line—activation border; solid line—mean fluorescence intensity of wtMpl/GFP double positive cells). (B) Human erythroid leukemia (HEL) cells were transduced with retroviral vectors encoding dnMpl, cd-dnMpl or GFP as control. wtMpl expressing 32D cells (100% positive) were mixed with untransduced, GFP control, dnMpl or cd-dnMpl expressing HEL cells in different ratios (1:1, 1:2, 2:1). Cells were co-cultured in murine Thpo supplemented medium (6ng/mL). The percentage of 32D cells three and six days after co-culture was measured. In the absence of dnMpl or cd-dnMpl wtMpl/32D cells grew faster than HEL cells. But in the presence of dnMpl (1:1, 1:2 and 2:1) or cd-dnMpl (1:2) wtMpl/32D cells stopped proliferating and were diminished over time in the culture. (C) Blood counts of mice transplanted with dnMpl, cd-dnMpl or GFP control transduced wildtype Lin- BM cells six and twelve weeks after transplantation (*p<0.05, ***p<0.005 Student’s t-test, n = 4–5). One of the cd-dnMpl mice had to be killed at 12 weeks due to the severe anemia.

To test whether dnMpl exerts its effects by a cell intrinsic or extrinsic mechanism, we performed a set of *in vitro* experiments using cell line models. First, we investigated whether dnMpl expression interfered with wtMpl receptor signaling when co-expressed on the same cell. The myeloid cell line 32D was engineered to grow Thpo dependent by retroviral expression of wtMpl from the phosphoglycerate kinase (PGK) promoter. wtMpl/32D cells were transduced with the SF91.dnMpl.IRES.GFP retroviral vector to obtain a mixture of wtMpl single and wtMpl/dnMpl double positive cells. dnMpl expression from the SFFV promoter was stronger than the expression of wtMpl from the PGK promoter which mimics the *in vivo* situation. In an attempt to exclude heterodimerization of dnMpl with the wtMpl and, therefore, prevent the possibility of dnMpl monomers to block the activity of endogenous wtMpl, we also expressed a constitutive-dimerized (cd) form of dnMpl. To achieve covalent dimerization, we introduced an S to C mutation at amino acid 368 of the dnMpl extracellular domain. This mutation results in a stable dimerized conformation via a disulfide bridge of two Mpl receptor chains [32]. wtMpl/dnMpl and wtMpl/cd-dnMpl cells were then stimulated by Thpo and analyzed for STAT5 and ERK1/2 phosphorylation by flow cytometry. Strikingly, the expression of dnMpl but not cd-dnMpl inhibited wtMpl-induced STAT5 phosphorylation at low Thpo concentrations (2.5 and 5 ng/ml) and inhibition declined with higher doses (20 ng/ml and 50ng/ml; S4 Fig). This suggests that expression of dnMpl intrinsically inhibits wtMpl signaling at physiological Thpo concentrations by heterodimerization. However, at high Thpo doses this effect was abrogated (Fig 3A).

Next, we addressed the question whether dnMpl expression on one cell could interfere with Thpo/Mpl signaling on another cell. We developed dnMpl, cd-dnMpl and GFP expressing human erythro-leukemia (HEL) cell lines and mixed these cell lines with Thpo-dependent wtMpl/32D cells at different ratios (1:1, 1:2, 2:1). These cultures were then supplemented with 6ng/mL Thpo. When co-cultured with untransduced or GFP control transduced HEL cells wtMpl/32D cells grew faster and outcompeted the HEL cells. When challenged with dnMpl/HEL cells, the wtMpl/32D cells stopped proliferating and were diminished in the culture over time independent of the starting ratio (Fig 3B). A similar effect was observed with cd-dnMpl/HEL cells, however, to a lesser extent. These experiments demonstrated that the systemic presence of dnMpl on non-target cells interfered with wtMpl signaling on other cells by competition for Thpo binding.

As final experiment we performed BM transplantations of cd-dnMpl transduced BM cells into lethally irradiated wt mice and compared to the transplantation of dnMpl and GFP control transduced BM (experiment 3). Peripheral blood cell analysis six and twelve weeks post transplantation revealed low platelet counts at both time points (Fig 3C) in the cd-dnMpl transplanted group equal to the dnMpl group. The hematocrit again remained unchanged, but cd-dnMpl and dnMpl mice showed significantly reduced leukocyte numbers compared to the GFP control mice (p<0.05, dnMpl n = 5, cd-dnMpl n = 4; Fig 3C).

### Mice transplanted with dnMpl transduced BM have low LSK cell numbers

**Fig 4:**
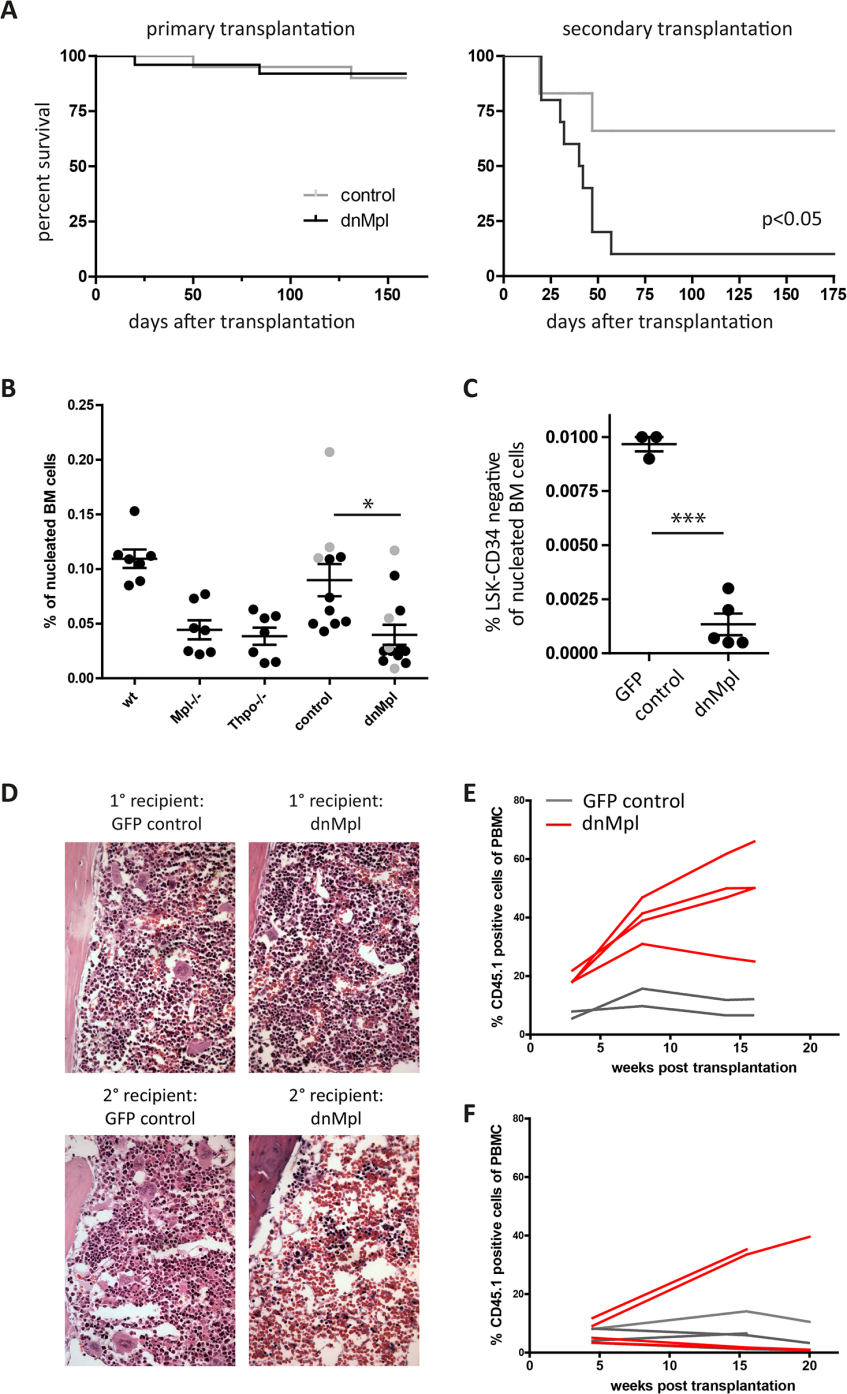
Impact of dnMpl expression on the HSC compartment. (A) Survival curve of primary transplanted mice of the dnMpl or the control groups (experiment 1–3, dnMpl n = 25, control n = 20). Of experiment three, the BM was transplanted into two secondary recipients each (3 dnMpl donors, 2 cd-dnMpl donors, 3 GFP control donors). There was no difference in survival in primary recipients but a significantly reduced survival of secondary recipients (10 dnMpl recipient mice and 6 control recipient mice, p<0.05). (B) Percentage of LSK cells in primary recipients (dnMpl and trCD34 control black filled circles, cd-dnMpl and GFP control in grey filled circles) or in steady state hematopoiesis (wt, *Mpl-/-*, *Thpo-/-*). (*p<0.05, Students t-test). (C) Percentage of LSK-CD34 negative cells in primary recipients of GFP control (n = 3) or dnMpl mice (n = 5) (***p<0.005, Students t-test). (D) Histological analysis of the BM of dnMpl and control transplanted mice. Primary (1°) dnMpl chimeric mice had reduced numbers and smaller megakaryocytes similar to *Mpl-/-* mice. Secondary dnMpl recipient mice (2°) had a hypocellular BM in agreement with the symptoms of BM failure. BM sections were Hematoxylin/Eosin stained and microscopic images were taken at 200x magnification. (E) CD45.2 wildtype C57Bl/6 mice were transplanted with dnMpl, cd-dnMpl or GFP control transduced CD45.2 wildtype lin- BM cells (experiment 3). 19 weeks after the first transplantation, two dnMpl, two cd-dnMpl and two GFP mice were infused with a second graft of 2x10^7^ CD45.1 whole BM cells without further conditioning. The chimerism of CD45.1 in the blood leukocytes was analyzed over a period of 16 weeks. dnMpl conditioned mice allowed the engraftment of a second wt graft without further conditioning. (F) In experiment 5, CD45.2 wildtype C57Bl/6 mice were transplanted with dnMpl or GFP control transduced CD45.2 wildtype lin- BM cells, however, with intended lower chimerism. 19 weeks after the first transplantation mice were given a second graft of 2 x10^7^ CD45.1 whole BM cells without further conditioning. The chimerism of CD45.1 in the blood leukocytes was analyzed over a period of 20 weeks. While the two mice exhibiting the highest percentage of dnMpl expression (4 and 14%) allowed stable engraftment of the second graft, the mice with < 1% dnMpl expression in the periphery failed to do so and presented with the low engraftment levels similar to the four GFP control mice.

The survival of primary recipients of dnMpl or control transduced BM was normal (dnMpl n = 25, control n = 20). After serial transplantation, however, secondary dnMpl recipients died due to graft failures starting from three weeks after transplantation while survival of secondary control mice was not impaired (experiment 3, dnMpl n = 10, control n = 6, Fig 4A).

The low secondary engraftment potential of BM cells of dnMpl mice suggested HSC defects. Therefore, we analyzed the HSC-enriched compartment defined by the lineage marker-negative, Sca1 and c-kit positive (LSK) cell surface phenotype, (referred to as hematopoietic stem and progenitor cells (HSPC)) of dnMpl and control mice by flow cytometry. Primary recipients transplanted with dnMpl expressing BM cells had reduced numbers of LSK cells (0.039+/-0.009% of the nucleated BM, n = 13) compared to control transplanted mice (0.089+/-0.015, n = 11, p-value = 0.0032; Fig 4B, S5 Fig) and similar to the levels found in *Mpl-/-* and *Thpo-/-* mice in steady state hematopoiesis (0.045+/-0.021%, n = 14) as also previously reported [33]. The LSK cell level was similarly reduced in mice transplanted with the cd-dnMpl transduced BM cells (0.052+/-0.047%, Fig 4B, filled grey circles). Also, the more HSC enriched population of CD34-/LSK cells was significantly reduced in dnMpl transplanted mice (Fig 4C).

Histopathological analysis of the BM revealed impaired megakaryopoiesis with reduced numbers and smaller megakaryocytes (MK) in primary and secondary dnMpl recipients similar to *Mpl-/-* mice, while the BM of GFP control transplanted mice was normal (Fig 4D, S6 Fig). Also, in secondary dnMpl mice the BM was hypocellular confirming the graft failures in these mice.

As dnMpl exposed mice presented with HSC defects, we investigated whether the stem cell niche in these mice would allow the engraftment of a second wt BM graft. To test this, 2x10^7^ total CD45.1-congenic BM cells were injected into two dnMpl, two cd-dnMpl and two GFP control mice 19 weeks after the first transplantation without any further preconditioning. All four injected mice of the dnMpl groups showed high blood donor chimerism, which increased with time (47.8+/-15.7% at the end of the observation time) while the two injected GFP control mice had only low levels of blood chimerism (Fig 4E). We confirmed our observation in one further experiment in which mice had lower levels of transgenic cells (Fig 4F). Flow cytometry analysis also revealed high BM chimerism in the dnMpl conditioned mice compared to GFP controls (S7 Fig). In summary, the impaired survival of secondary dnMpl recipients, the reduced numbers of LSK cells and the defective MK maturation and platelet production suggested a Thpo/Mpl-deficient situation in dnMpl transplanted mice. Although unmodified hematopoietic cells were co-transplanted, these cells did not rescue the dnMpl-induced BM failures. Therefore, the dnMpl expression on a subset of hematopoietic cells impaired the signaling by the endogenous Mpl receptor also in the untransduced cells.

### LSK cells in dnMpl expressing mice have a deregulated gene expression profile

**Fig 5:**
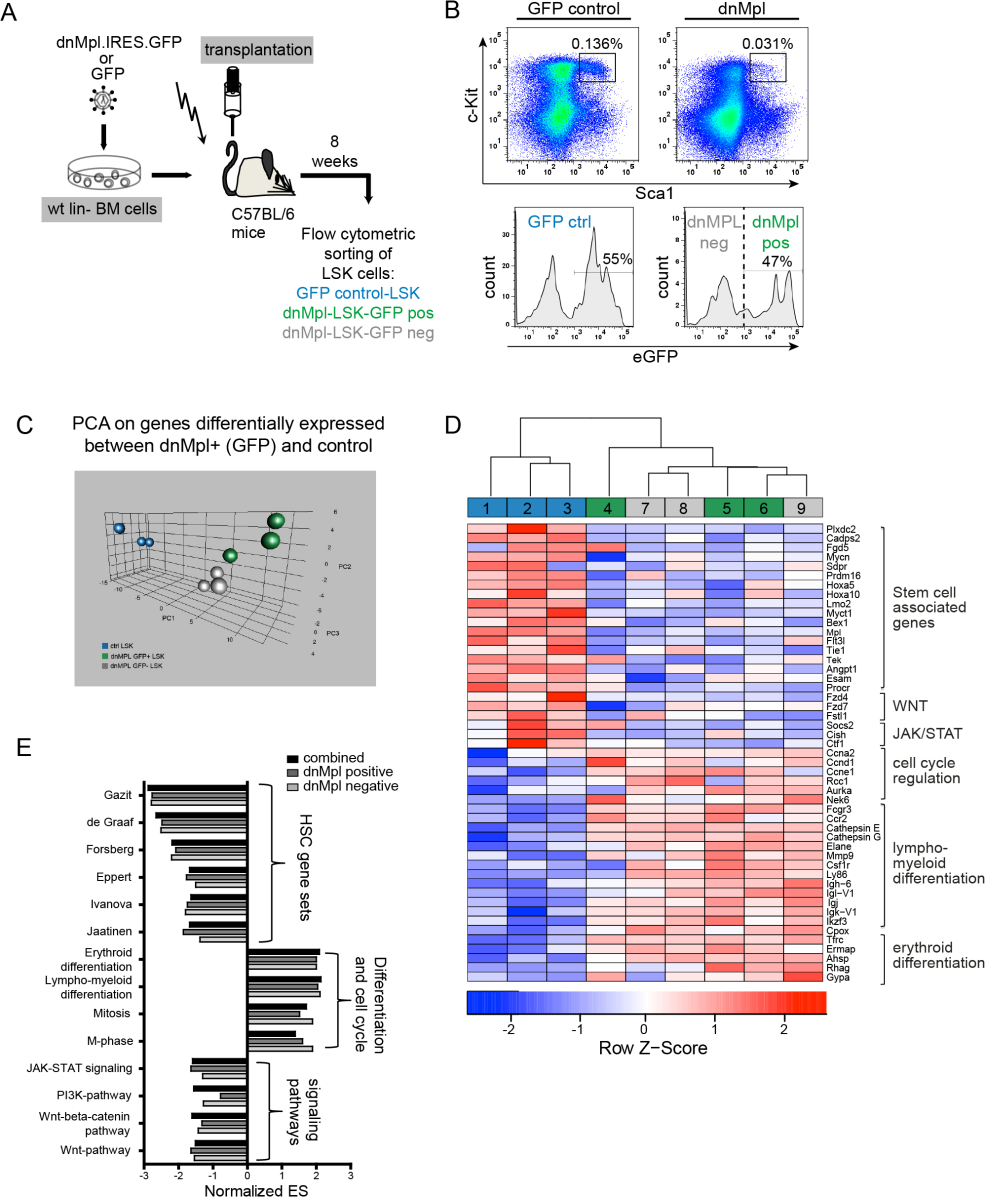
Gene expression analysis of LSK cells from dnMpl mice. (A) Schematic picture of the performed experiment. Wildtype Lin- BM cells were transduced with either GFP or dnMpl.IRES.GFP expressing vectors and transplanted into lethally irradiated wt recipients. Eight weeks after transplantation LSK cells were sorted using flow cytometry. (B) Representative FACS blots of the BM cells of GFP control and dnMpl.IRES.GFP transplanted mice. In control mice GFP positive and negative LSK cells were pooled, whereas for dnMpl.IRES.GFP mice GFP positive (47%) and negative (53%) LSK cells were separately subjected to transcriptome analysis. (C) Principal component analysis (PCA) on genes differentially expressed between dnMpl-GFP+ and control LSK cells. (D) Heatmap of selected genes found to be deregulated in the gene expression analysis. dnMpl positive and negative LSK cells from the same mice (dnMpl pos (4–6) and neg (7–9)) were compared to the expression in LSK of GFP control transplanted mice (control 1–3). (blue: donwregulated genes, red: upregulated genes). (E) Gene set enrichment analysis (GSEA) of dnMpl versus GFP control transplanted mice. The expression matrix of dnMpl positive and dnMpl negative cells from the same mice were either used in combination or separately for the GSEA. The normalized enrichment scores (NES) of gene sets are displayed that are significantly enriched in the dnMpl phenotype. Most of the gene sets were part of the gene set collection of the GSEA tool and depicted based on the following publications [13,15,34,35,46,47,54] or based on the KEGG database.

To further elucidate the molecular effects of dnMpl expression on HSPC, we performed microarray analysis on LSK cells from dnMpl and GFP control mice sorted eight weeks after transplantation (Fig 5A). In the transplanted mice approximately 50% of the LSK cells were dnMpl positive. For comparison of dnMpl expressing versus non expressing cells we sorted dnMpl positive and negative LSK cells from the same mice (Fig 5B). We determined which genes were differentially expressed between dnMpl+ and ctrl LSK cells. Strikingly, hierarchical clustering and PCA analysis on these genes revealed that gene expression in dnMpl+ and dnMpl- LSK cells in mice transplanted with dnMpl-cells was very similar, as they clustered together, and were clearly distinct from the control mice (Fig 5C and 5D). This indicated that expression of dnMpl in the bone marrow impacted transgene positive and negative LSK-cells in a similar way.

**Fig 6:**
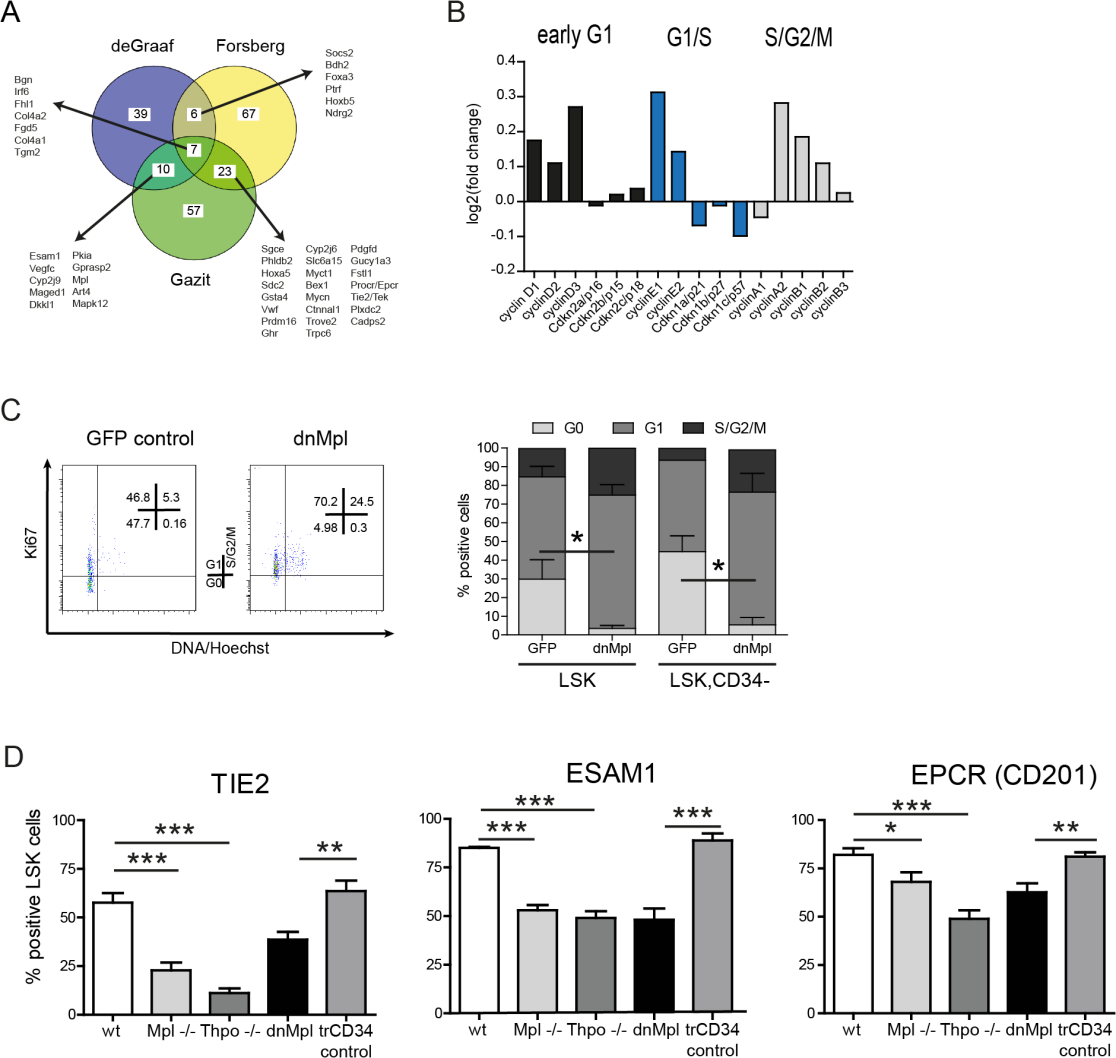
Cell cycle analysis and cell surface molecule expression on LSK cells in dnMpl mice. (A) Venn diagram showing the overlap of the leading edge genes from the Gene Set Enrichment Analysis comparing our dataset with the data of [15,34,54]. (B) Expression of essential players in the cell cycle control. Displayed are the log2(fold-change) values determined based on the microarray analysis in comparison to control. (C) Cell cycle analysis of LSK cells of dnMpl and GFP control transplanted mice. Total BM cells were stained and gated for the LSK or LSK, CD34- cell population. Cell cycle status was determined by staining with Ki-67 and Hoechst 33342. A representative example of the flow cytometric analysis is shown (left picture) and the results summarized (right graph). Significantly less LSK and LSK, CD34- cells are within the G0 phase of the cell cycle (*p<0.05, n = 3). (D) Wild-type mice were transplanted with dnMpl or truncated (tr)CD34 (as control) expressing BM cells. At 16 weeks post transplantation total BM cells were stained for LSK cells, each BM sample was then splitted into three subsamples to stain for the different surface molecules: TIE2, EPCR (CD201), ESAM1. As controls, cell surface expression was also detected in untransplanted wt, *Mpl-/-* and *Thpo-/-* mice. The difference between the percentages of surface marker positive cells was significant, as indicated: *p<0.05, **p<0.01, ***p<0.005. (wt, *Mpl-/-*, *Thpo-/-*: n = 6; dnMpl, trCD34: n = 9).

We next sought to analyze to which cellular pathways and processes the affected genes belonged to. Gene set enrichment analysis (GSEA) of expression data from dnMpl+ and dnMpl- LSK cells of the same mice was performed separately and compared to LSK cells from control transplanted mice. In agreement with the hierarchical clustering and PC analyses similar gene set enrichment results were obtained with the dnMpl+ and dnMpl- expression profiles (Fig 5E, S8 Fig). Consistent with the Mpl-deficient phenotype, the two main downstream signaling pathways JAK-STAT and PI3K/AKT were downregulated. As another important signaling pathway for HSC we found that Wnt-signaling was downregulated in the dnMpl-mice. Most importantly, LSK cells from dnMpl-mice displayed a striking loss of hematopoietic stem-cell associated expression signatures, e.g. the signature of long term HSC [34] and human CD34+HSC [35], and the Thpo-responsive signature of HSC [15]. Gene expression analysis confirmed the induction of stem cell defects by dnMpl expression. This is in agreement with the role of Thpo/Mpl signaling in HSC maintenance and the loss of this signaling will inevitably cause HSC defects [6,33,36]. By comparing the leading edge genes of the aforementioned gene set enrichment analysis we detected substantial overlap of HSC-associated genes that are also deregulated in our dataset (e.g. *Esam1*, *Plxdc2*, *Tie2*, *Prdm16*, *Procr/Epcr*, *MycN*, *Fhl1*, *HoxA5*, *Socs2;* Fig 6A).

### Loss of Mpl-signaling increases HSPC cycling and reduces the expression of stem cell-associated surface molecules

Gene expression in dnMpl HSPC was consistent with increased cell cycle progression. Expression levels of D- and E-Cyclins were increased (Fig 6B). Expression levels of the cyclin-dependent kinase inhibitors Cdkn1a/p21 and Cdkn1c/p57 were decreased while Cdkn2c/p18 was 50% upregulated (Fig 6B). Cdkn1c/p57 is a known target for Thpo-induced HSC quiescence [36] and Cdkn1a/p21 knockout mice have a defect in stem cell maintenance [37]. In contrast, low Cdkn2c/p18 levels were reported to increase HSC self-renewal [38]. To confirm the increased cell cycle status of HSPC we performed Hoechst/Ki67 staining in LSK cells of dnMpl and control vector transplanted mice 23 weeks after transplantation (Fig 6C). In dnMpl mice, a significantly increased number of LSK and LSK, CD34- cells were in the G1/S/G2 phases of the cell cycle and less in G0 (3.5 ± 0.9 versus 29.8 ± 6.1 in LSK and 5.4 ± 3.3 versus 44.5 ± 7 in LSK, CD34- cells n = 3, p = 0.013 and p = 0.018, respectively), confirming increased cell cycle activity in the population enriched for HSC, probably causing progressive HSC exhaustion.

To validate the results from the gene expression analysis on the reduced expression of surface markers in dnMpl exposed LSK cells, we investigated protein expression of the cell surface molecules by flow cytometry. We analyzed TIE2, the receptor for Angiopoietin-1, which was reported to be linked to Mpl expression on long-term HSC [36], EPCR (endothelial protein C receptor, CD201) and ESAM1 (endothelial cell-selective adhesion molecule). The latter two are surface markers on HSC with long-term repopulating capacity [39–41]. A significantly smaller proportion of LSK cells in dnMpl mice expressed these markers compared to wt or control transplanted animals (Fig 6D) and also at lower levels as indicated by the reduced fluorescence intensity (S9 Fig). A similar decrease in surface marker expression was observed in LSK cells from *Mpl-/-* and *Thpo-/-* mice (Fig 6D). The alteration of gene expression in cells with the HSC-associated phenotype in dnMpl mice was, therefore, also accompanied by decreased expression of important cell surface receptors that are thought to be involved in the sensing of signals in the BM niche.

### Comparison of the gene expression profiles in LSK cells of dnMpl mice with CD34+ cells of aplastic anemia patients

**Fig 7:**
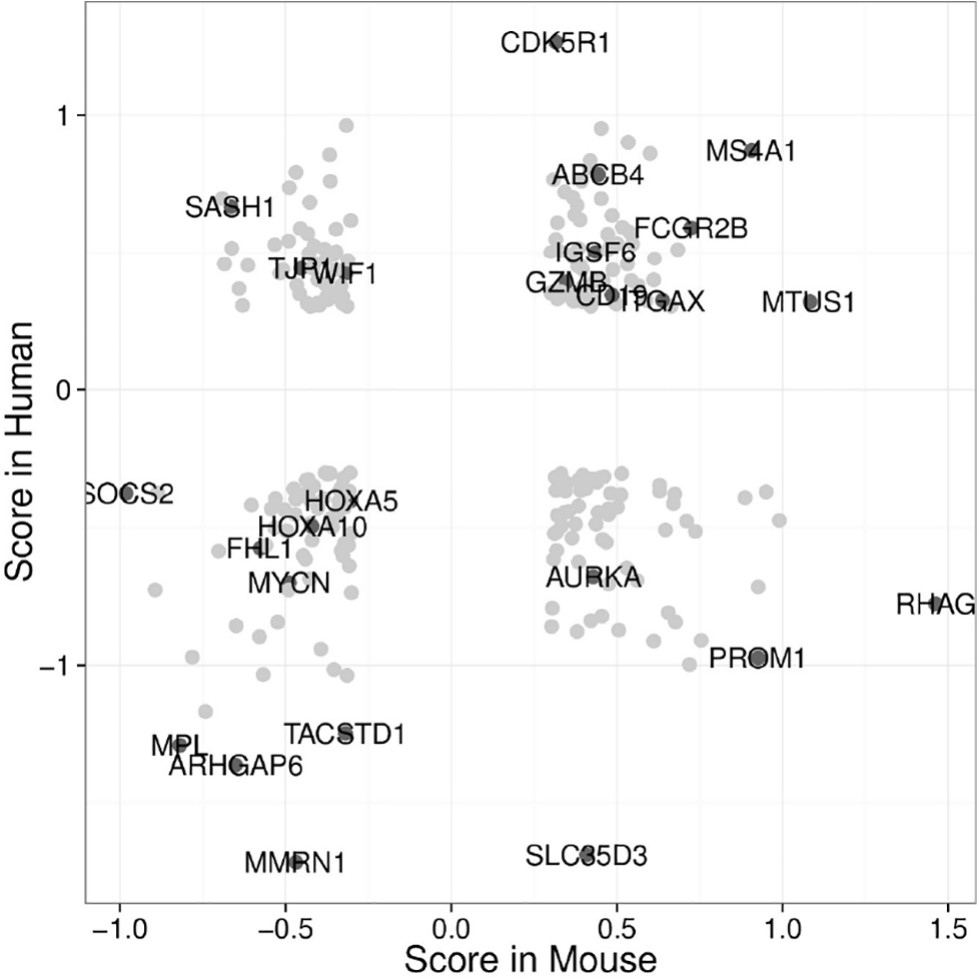
Comparison of dnMpl LSK cells and of CD34+ cells of aplastic anemia patient. (A) Expression scores of each gene in murine dnMpl mice and human RC and SAA patients CD34+ cells were compared. Negative scores in both cases (lower left quadrant) reflect the downregulation of genes typically associated with a healthy phenotype. Differentially expressed genes (lower right and upper left quadrant) may reflect species/disease differences. Score: (expression sample–expression control)/(SD sample + SD control). Gene lists referring to each quadrant in the supplements.

Defects in Mpl signaling have been linked to familial aplastic anemia [42] and CAMT. Having established that inhibition of Mpl-signaling by dnMpl causes progressive BM-failure in mice we sought to analyze whether there was an overlap that would indicate functional impairment of Mpl signaling in human bone marrow failure syndromes. We therefore compared the gene expression profile of dnMpl LSK cells with expression profiles from CD34+ cells of patients with severe aplastic anemia (SAA) and refractory cytopenia (RC) published by Fischer and colleagues [43]. Human CD34+ cells are ~100-times less enriched for LT-HSC than murine LSK cells [44]. However, information of the gene expression in HSC of human aplastic anemia patients is difficult to obtain due to the low number of HSC in this disease. Also, in contrast to our mouse model, in humans SAA are mostly caused by autoimmune attack of CD34+ cells and gene expression analysis by Fisher and colleagues confirmed this (upregulated genes in immune and stress response and cell death) [43]. In our analysis we focused on gene sets associated with hematopoietic progenitors and stem cells. GSEA suggested that the signature in RC/SAA CD34+ cells was characterized by a severe loss of stem cell associated genes and an upregulation of genes that indicate lympho-myeloid differentiation, very similar to the effects seen upon expression of dnMpl (Fig 7A). Strikingly, MPL itself was one of the most downregulated genes in both SAA and RC with a mean log_2_FC of -3.5 (p<10^−7^, S11 Fig). In contrast to dnMpl LSK cells, cell cycle promoting genes were downregulated in CD34+ HSC from RC and SAA. By comparing gene deregulations based on scores which correlate the expression value to their respective controls in the murine dnMpl and human SAA/RC expression profiles, we identified genes with a similar positive and negative regulation (Fig 7A, S1–S4 Tables). Although there was no overall correlation between human and murine scores, important stem cell genes were similarly deregulated in both profiles.

## Discussion

By deleting the intracellular signaling domains we generated a dominant-negative Mpl receptor that was presented on the cell surface. Retroviral expression of dnMpl in the BM of wt mice induced thrombocytopenia and a progressive loss of HSC. The number of LSK cells in the BM of primary dnMpl recipients was threefold reduced compared to control transplanted mice and the BM of primary dnMpl mice was unable to engraft in secondary recipients demonstrating HSC defects in these mice. Functional HSC defects were further supported by gene expression analyses which demonstrated a loss of HSC-typical signatures in LSK cells of dnMpl mice.

Since our experiments were based on gammaretroviral transduction with efficiencies of about 50%, the recipient mice received a mixed pool of transduced and untransduced cells. However, untransduced cells did not rescue the HSC defects nor did they out-compete dnMpl expressing hematopoietic cells. Therefore, there must be a bystander effect from dnMpl expressing cells on non-expressers. In *in vitro* experiments we demonstrated that dnMpl expression on a subset of cells can interfere with Thpo/Mpl signaling on other cells. Furthermore by the use of the constitutive-dimerized dnMpl we proved that the main mechanism of inhibition was the competition for Thpo binding and not the formation of heterodimers with the endogenous wtMpl. These observations were further strengthened by the clustered gene expression profiles in dnMpl positive versus negative LSK cells from the same mice. As dnMpl lacks the intracellular domains important for internalization after ligand binding, we did not observe decreased Thpo levels in the blood. Therefore, thrombocytopenia and HSC defects were induced independent of the Thpo levels. As dnMpl was anchored to the cell membrane, receptor competition for Thpo must have taken place in the direct neighborhood of the affected cells, namely competition of dnMpl expressing hematopoietic progenitors and differentiated cells with the megakaryocytes and HSC in the BM.

A truncated Mpl isoform from the endogenous Mpl locus was described and is shared between mouse and human. This truncated Mpl lacks the transmembrane domain, therefore is soluble but remains intracellular. It was demonstrated that this isoform downregulates Mpl protein levels. This effect was mediated by a unique 30 aa sequence at the C-terminus of the protein [45] and by addition of cathepsin inhibitors this effect could be abolished. However, in our study, this C-terminal residue is absent, therefore the mechanism as described will not have played a role in our model.

The Thpo/Mpl signaling-deficient situation in dnMpl mice was demonstrated by the downregulation of important genes in the JAK/STAT and PI3K/AKT pathways downstream of Mpl in the HSC-enriched population, detected in the gene expression arrays. Furthermore, HSC stemness gene expression signatures were negatively enriched to the dnMpl expression profile [34,35,46,47]. This also included the Wnt pathway, which is essential for proper development of the HSC [48] and HSC regeneration [49]. Furthermore, the LSK and LSK, CD34- cells of mice expressing dnMpl were less often found in the G0 phase of the cell cycle and expressed higher levels of D and E cyclins, confirming a higher HSPC turnover which will have led to HSC exhaustion and differentiation [50]. The role of Thpo/Mpl signaling to maintain HSC quiescence is established [33,36]. Comparison of differential gene expression in the dnMpl LSK cells with that in human RC/SAA CD34+ cells identified the same stem cell genes to be downregulated. In the murine model, we confirmed downregulation of TIE2, EPCR and ESAM1, all of which have assumed functions in HSC maintenance, but only TIE2 has been linked to Thpo/Mpl-signaling. It was reported that Mpl inhibition by *in vivo* application of AMM2 antibodies resulted in decreased Tie2 expression, and Mpl activation by recombinant Thpo increased Tie2 expression [18]. EPCR was shown to be expressed on the majority of side population cells (>90%) and to mark the cell population with robust hematopoietic reconstitution capacity [39]. Likewise, high expression of ESAM1 defines LSK cells with high long-term reconstitution capacity, especially under stress conditions [41]. Although the impact of EPCR and ESAM1 is not well understood on the molecular level, they are important markers for LT-HSC. Our data demonstrate a correlation of their expression with Thpo/Mpl signaling.

In a reverse model, we have previously shown that *Mpl* gene therapy in the *Mpl-/-* mouse model was able to recover prior defective stem cells [13]. Together with the data presented in this study, it highlights that HSC properties are rather plastically modulated by Thpo/Mpl signals and, therefore, activation of Mpl signaling may regenerate defective HSC in aplastic anemias. Indeed, treatment of aplastic anemia patients with thrombopoietin mimetics, such as eltrombopag and romiplostim, have demonstrated clinical efficacy [51,52]. However, side-effects of long-term treatment of aplastic anemia patients with direct Mpl activators such as the expansion of preleukemic clones have still to be considered [53]. Therefore, the analysis of the molecular changes in HSC after inhibition of Thpo/Mpl signaling in the adult mouse as performed in our study adds to our understanding of how Thpo/Mpl signals regulate HSC quiescence and maintenance. The better characterization of Thpo/Mpl-dependent genes and pathways will thereby foster the identification of therapeutic targets.

## Supporting Information

S1 Fig Cell surface expression of wtMpl, dnMpl and trCD34. (A) 32D cells were transduced with HA-wtMpl or HA-dnMpl encoding vectors to compare surface protein expression of wtMpl and dnMpl by flow cytometry. Two examples with either high or low transduction efficiency are depicted. wtMpl and dnMpl are equally expressed on the cell surface. (B) Exemplary FACS blots of leukocytes stained for the presence of the HA-tag of mice transplanted with dnMpl or trCD34 transduced lineage negative BM cells. wtMpl, dnMpl and trCD34 protein expression was detected by staining of the HA-tag with a FITC conjugated monoclonal antibody (1:100 diluted, Roche Diagnostics, Mannheim, Germany).
(PDF)

S2 Fig dnMpl is expressed on all blood cell types. Transgene expression in the different blood lineages: C57Bl/6 Lin- BM cells were transduced with dnMpl or trCD34 and transplanted into lethally irradiated C57Bl/6 recipients. Shown is the average percentage of transgene positive cells (mean±SD, n = 4) of each cell lineage based on the staining of leukocyte (CD3, B220, CD11b) or whole blood cells using the aHA-FITC antibody sixteen weeks post transplantation. Both transgenes were expressed among the different blood cell types.
(PDF)

S3 Fig dnMpl does not interfere with lymphoid or myeloid differentiation. C57Bl/6 Lin- BM cells were transduced with dnMpl or trCD34 and transplanted into lethally irradiated C57Bl/6 recipients. These mice were monitored for their T-cell, B-cell and myeloid reconstitution six, eight and sixteen weeks post transplantation. Blood samples were stained with anti-CD3, anti-B220 and anti-CD11b antibodies to identify T-cells, B-cells or myeloid cells, respectively. The average percentage of each cell type at the given time points is shown (Mean±SD, n = 4). No differences in lymphoid and myeloid recovery between the dnMpl and the control groups were observed.
(PDF)

S4 Fig Inhibition of wtMpl signaling by dnMpl is abrogated at high mThpo doses. wtMpl expressing 32D cells were transduced with dnMpl.IRES.GFP, constitutive dimerized (cd)-dnMpl.IRES.GFP or GFP encoding vectors to establish cultures with single and double positive cells. Cells were starved of any cytokine stimuli for 16 hrs and stimulated with 20 or 50 ng/mL mThpo for 15 minutes the next day. Unstimulated (negative control) and stimulated cells were fixed and permeabelized to allow intracellular staining of phosphorylated signaling molecules. Anti-phosphoERK1/2 or phosphoSTAT5 antibodies conjugated to Alexa Fluor 647 (BD Biosciences) were used. Shown are histogram overlays of pERK1/2 and pSTAT5 activation from wtMpl/GFP negative cells and wtMpl/GFP, wtMpl/dnMpl, wtMpl/cd-dnMpl double positive cells. Inhibition of wtMpl-signaling which was observed with low mThpo doses is absent when high mThpo doses (20 and 50 ng/ml) were applied.
(PDF)

S5 Fig Flow cytometric analysis of the LSK compartment. BM cells were pre-gated for lineage marker negative cells and then analyzed for the expression of Sca1 and c-kit. The contribution of LSK cells in the BM was reduced in dnMpl chimeric mice. Exemplary FACS blots of a trCD34 control transplanted and dnMpl mouse, as well as of untransplanted wildtype, Mpl-/-, and Thpo-/- mice are depicted.
(PDF)

S6 Fig Bone marrow histology of untransplanted controls. Hematoxylin/Eosin stained bone marrow section of an untransplanted wildtype and Mpl-/- mouse. Mpl-/- BM contained lower numbers of megakaryocytes with lower ploidy.
(PDF)

S7 Fig BM donor chimerism after the transplantation of the second graft into dnMpl or GFP control mice. CD45.2 wildtype C57Bl/6 mice were transplanted with dnMpl or GFP control transduced CD45.2 wildtype lin- BM cells. 16 weeks after the first transplantation, dnMpl and GFP mice were infused with a second graft of 2x107 CD45.1 whole BM cells without further conditioning. After further 17 weeks, mice were sacrificed and the contribution of the second BM transplant was analyzed based on the CD45.1 cell surface expression by flow cytometry. dnMpl mice allowed the engraftment of CD45.1 donor cells long term as indicated by the high BM chimerism compared to the GFP control mice.
(PDF)

S8 Fig Exemplary Gene Set Enrichment blots of dnMpl versus GFP control mice analysis. Enrichment blots of different gene sets either enriched in the dnMpl or control phenotype. Supplied are the normalized enrichment score (NES), the nominal p-value, and the false discovery rate (FDR).
(PDF)

S9 Fig Protein expression of HSC surface marker TIE2, ESAM1 and EPCR (CD201). Representative samples of flow cytometric analyses demonstrate the reduced level of TIE2, ESAM1 and EPCR expression on LSK cells of mice transplanted with dnMpl cells in comparison to expression on LSK cells of control transplanted mice as measured by the reduced mean fluorescence intensity after staining with specific antibodies. (Dashed line–respective wildtype control; solid line–respective test phenotype).
(PDF)

S10 Fig Gene Set Enrichment Analysis of gene expression profiles in the aplastic anemia subtypes refractory anemia (RC) and Severe Aplastic Anemia (SAA). Blotted are the normalized enrichment scores (NES) of the Gene set enrichment analysis comparing RC and SAA or the both in combination with known gene sets as indicated.
(PDF)

S11 Fig Boxplot of Mpl expression in aplastic anemia patients (RC, SAA). The log2 fold change of MPL expression in refractory cytopenia (RC) and severe aplastic anemia patients (SAA) in comparison to CD34+ cells of healthy donors (ctrl) is shown[43].
(PDF)

S1 Table Gene list of murine and human gene expression score lower -0.3. The expression score is calculated as followed; (expression sample–expression control)/(SD sample + SD control), and the results ordered by the scores of the human genes. Expression scores lower -0.3 reflect the downregulation of the listed genes in both mouse and human.
(PDF)

S2 Table Gene list of murine and human gene expression score higher 0.3. The expression score is calculated as followed; (expression sample–expression control)/(SD sample + SD control), and the results ordered by the scores of the human genes. Expression scores higher 0.3 reflect the upregulation of the listed genes in both mouse and human.
(PDF)

S3 Table Gene list of murine gene expression score higher 0.3 and human gene expression score lower -0.3. The expression score is calculated as followed; (expression sample–expression control)/(SD sample + SD control), and the results ordered by the scores of the human genes. Expression scores higher 0.3 in human and lower -0.3 in mouse reflect the upregulation and downregulation of the listed genes, respectively.
(PDF)

S4 Table Gene list of murine gene expression score lower -0.3 and human expression score higher 0.3. The expression score is calculated as followed; (expression sample–expression control)/(SD sample + SD control), and the results ordered by the scores of the human genes. Expression scores higher 0.3 in mouse and lower -0.3 in human reflect the upregulation and downregulation of the listed genes, respectively.
(PDF)
