# Supplementary figures and images for: Inhibition of Thrombopoietin/Mpl Signaling in Adult Hematopoiesis Identifies New Candidates for Hematopoietic Stem Cell Maintenance
Source: PLoS One. 2015 Jul 6;10(7):e0131866. doi: 10.1371/journal.pone.0131866 (PMC4493002; doi:10.1371/journal.pone.0131866)

**A**

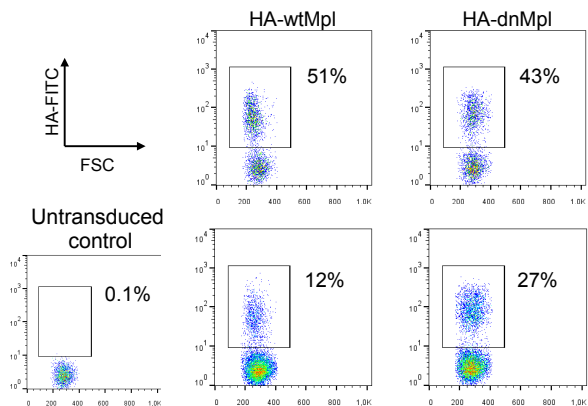

**B**

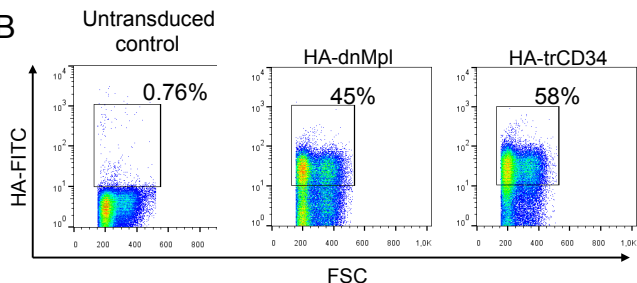

Supplement: S1 Fig — (A) 32D cells were transduced with HA-wtMpl or HA-dnMpl encoding vectors to compare surface protein expression of wtMpl and dnMpl by flow cytometry. Two examples with either high or low transduction efficiency are depicted. wtMpl and dnMpl are equally expressed on the cell surface. (B) Exemplary FACS blots of leukocytes stained for the presence of the HA-tag of mice transplanted with dnMpl or trCD34 transduced lineage negative BM cells. wtMpl, dnMpl and trCD34 protein expression was detected by staining of the HA-tag with a FITC conjugated monoclonal antibody (1:100 diluted, Roche Diagnostics, Mannheim, Germany). (PDF) [file pone.0131866.s001.pdf]

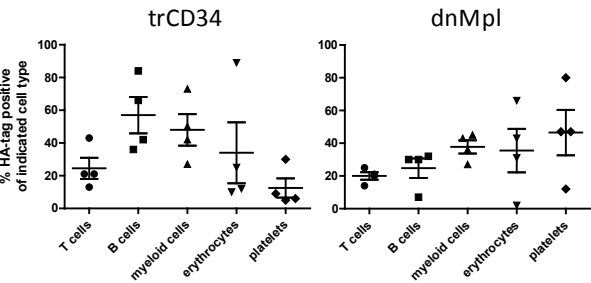

Supplement: S2 Fig — Transgene expression in the different blood lineages: C57Bl/6 Lin- BM cells were transduced with dnMpl or trCD34 and transplanted into lethally irradiated C57Bl/6 recipients. Shown is the average percentage of transgene positive cells (mean±SD, n = 4) of each cell lineage based on the staining of leukocyte (CD3, B220, CD11b) or whole blood cells using the aHA-FITC antibody sixteen weeks post transplantation. Both transgenes were expressed among the different blood cell types. (PDF) [file pone.0131866.s002.pdf]

### T cells

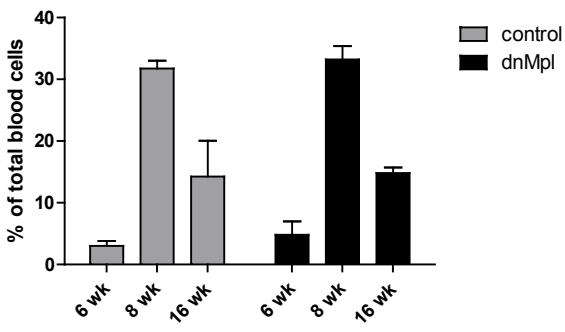

### B cells

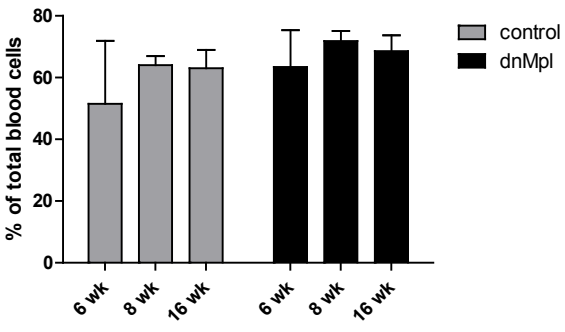

### myeloid cells

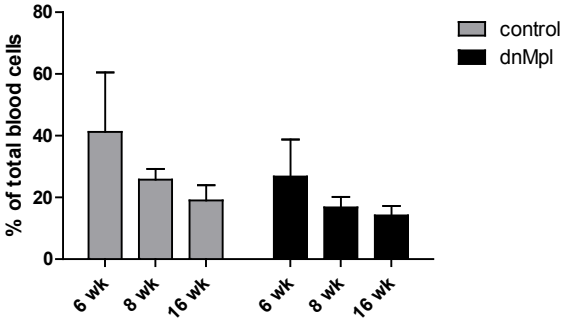

Supplement: S3 Fig — C57Bl/6 Lin- BM cells were transduced with dnMpl or trCD34 and transplanted into lethally irradiated C57Bl/6 recipients. These mice were monitored for their T-cell, B-cell and myeloid reconstitution six, eight and sixteen weeks post transplantation. Blood samples were stained with anti-CD3, anti-B220 and anti-CD11b antibodies to identify T-cells, B-cells or myeloid cells, respectively. The average percentage of each cell type at the given time points is shown (Mean±SD, n = 4). No differences in lymphoid and myeloid recovery between the dnMpl and the control groups were observed. (PDF) [file pone.0131866.s003.pdf]

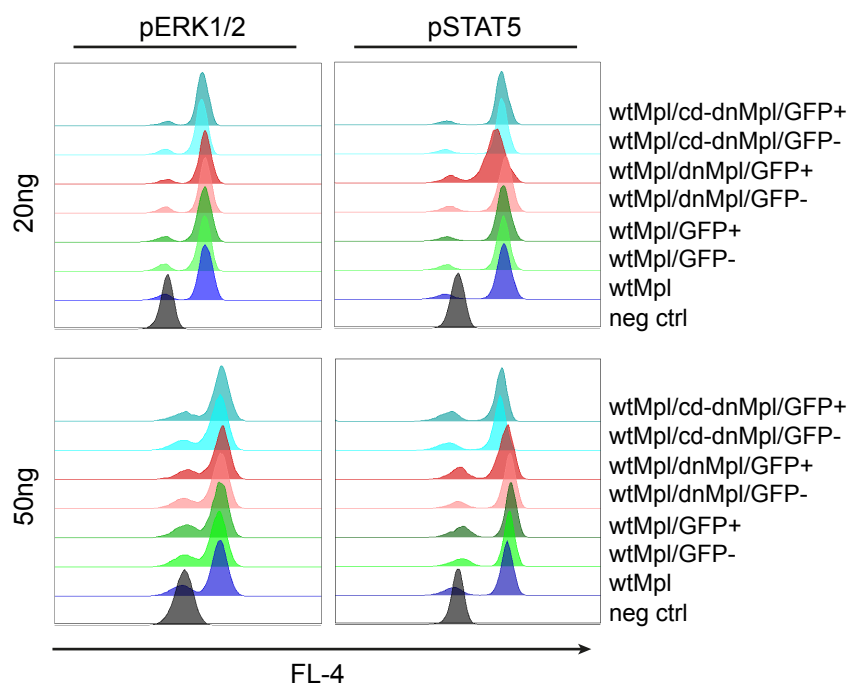

Supplement: S4 Fig — wtMpl expressing 32D cells were transduced with dnMpl.IRES.GFP, constitutive dimerized (cd)-dnMpl.IRES.GFP or GFP encoding vectors to establish cultures with single and double positive cells. Cells were starved of any cytokine stimuli for 16 hrs and stimulated with 20 or 50 ng/mL mThpo for 15 minutes the next day. Unstimulated (negative control) and stimulated cells were fixed and permeabelized to allow intracellular staining of phosphorylated signaling molecules. Anti-phosphoERK1/2 or phosphoSTAT5 antibodies conjugated to Alexa Fluor 647 (BD Biosciences) were used. Shown are histogram overlays of pERK1/2 and pSTAT5 activation from wtMpl/GFP negative cells and wtMpl/GFP, wtMpl/dnMpl, wtMpl/cd-dnMpl double positive cells. Inhibition of wtMpl-signaling which was observed with low mThpo doses is absent when high mThpo doses (20 and 50 ng/ml) were applied. (PDF) [file pone.0131866.s004.pdf]

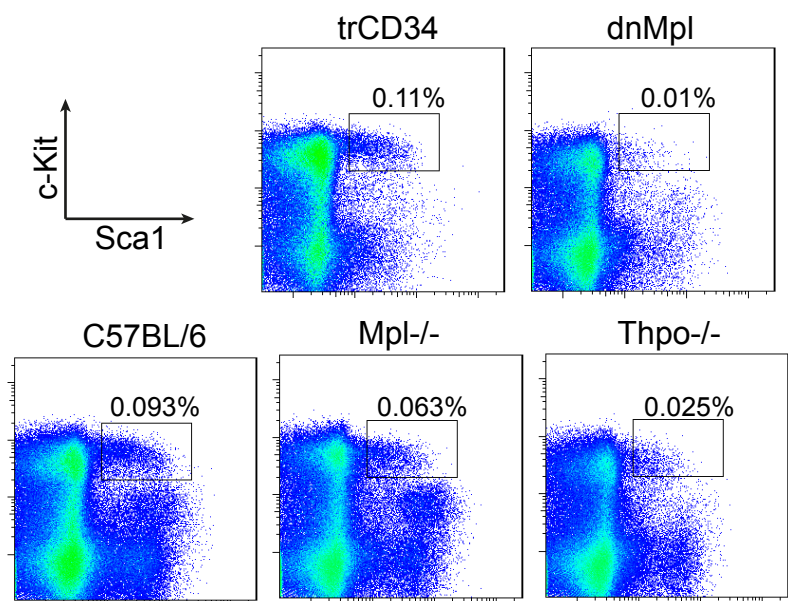

Supplement: S5 Fig — BM cells were pre-gated for lineage marker negative cells and then analyzed for the expression of Sca1 and c-kit. The contribution of LSK cells in the BM was reduced in dnMpl chimeric mice. Exemplary FACS blots of a trCD34 control transplanted and dnMpl mouse, as well as of untransplanted wildtype, Mpl-/-, and Thpo-/- mice are depicted. (PDF) [file pone.0131866.s005.pdf]

wildtype ctrl

Mpl<sup>-/-</sup> ctrl

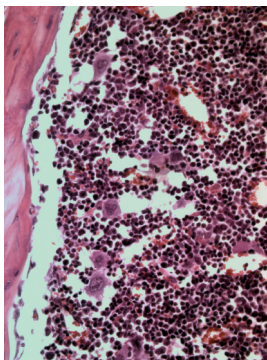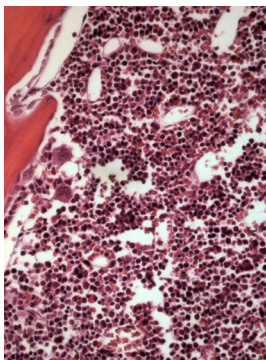

---

untransplanted

Supplement: S6 Fig — Hematoxylin/Eosin stained bone marrow section of an untransplanted wildtype and Mpl-/- mouse. Mpl-/- BM contained lower numbers of megakaryocytes with lower ploidy. (PDF) [file pone.0131866.s006.pdf]

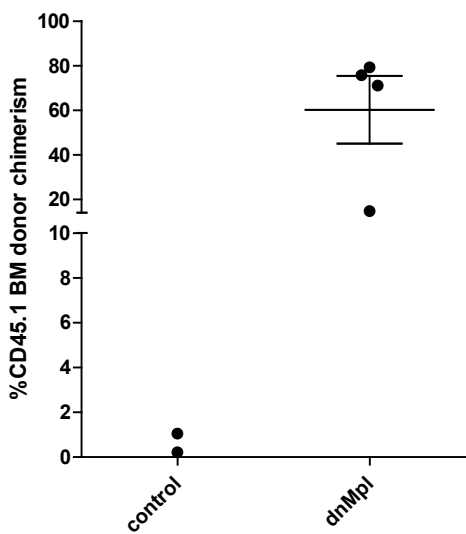

Supplement: S7 Fig — CD45.2 wildtype C57Bl/6 mice were transplanted with dnMpl or GFP control transduced CD45.2 wildtype lin- BM cells. 16 weeks after the first transplantation, dnMpl and GFP mice were infused with a second graft of 2x10^7^ CD45.1 whole BM cells without further conditioning. After further 17 weeks, mice were sacrificed and the contribution of the second BM transplant was analyzed based on the CD45.1 cell surface expression by flow cytometry. dnMpl mice allowed the engraftment of CD45.1 donor cells long term as indicated by the high BM chimerism compared to the GFP control mice. (PDF) [file pone.0131866.s007.pdf]

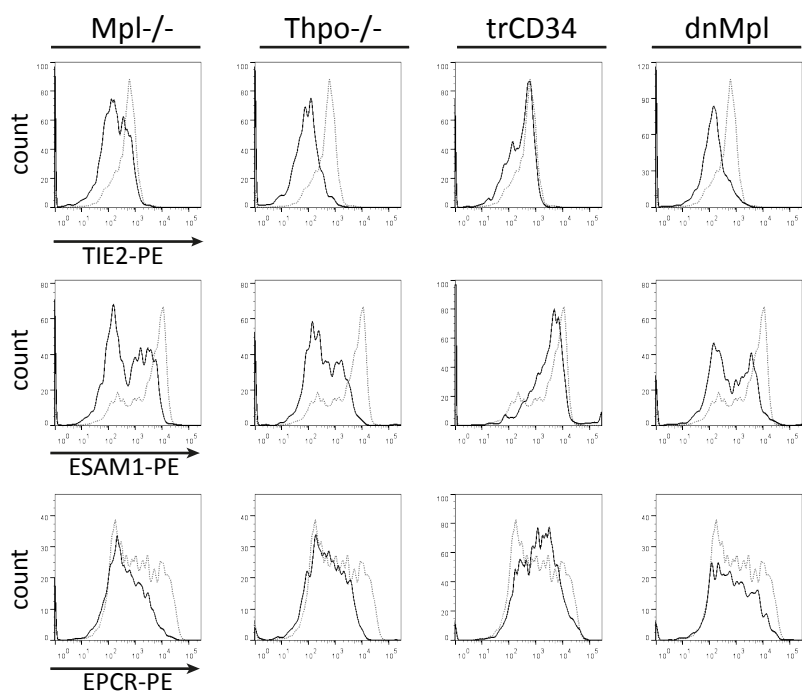

Supplement: S9 Fig — Representative samples of flow cytometric analyses demonstrate the reduced level of TIE2, ESAM1 and EPCR expression on LSK cells of mice transplanted with dnMpl cells in comparison to expression on LSK cells of control transplanted mice as measured by the reduced mean fluorescence intensity after staining with specific antibodies. (Dashed line–respective wildtype control; solid line–respective test phenotype). (PDF) [file pone.0131866.s009.pdf]

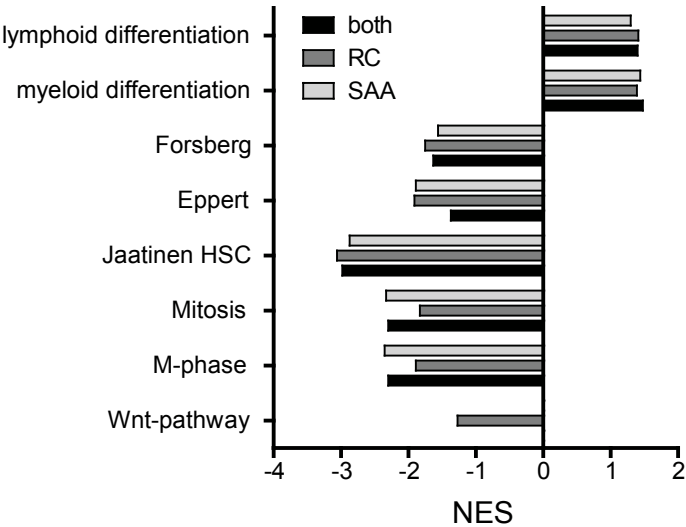

Supplement: S10 Fig — Blotted are the normalized enrichment scores (NES) of the Gene set enrichment analysis comparing RC and SAA or the both in combination with known gene sets as indicated. (PDF) [file pone.0131866.s010.pdf]

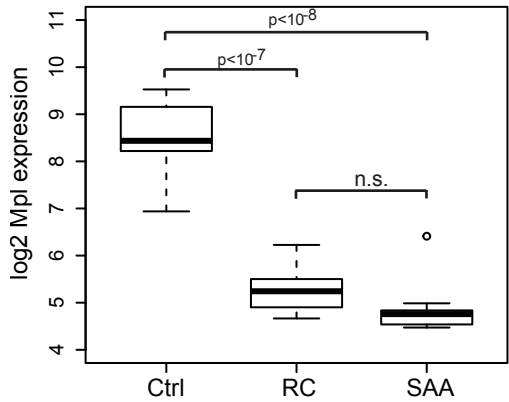

Supplement: S11 Fig — The log2 fold change of MPL expression in refractory cytopenia (RC) and severe aplastic anemia patients (SAA) in comparison to CD34+ cells of healthy donors (ctrl) is shown[43]. (PDF) [file pone.0131866.s011.pdf]
