# Supplementary material for: Inhibition of Thrombopoietin/Mpl Signaling in Adult Hematopoiesis Identifies New Candidates for Hematopoietic Stem Cell Maintenance
Source: PLoS One. 2015 Jul 6;10(7):e0131866. doi: 10.1371/journal.pone.0131866 (PMC4493002; doi:10.1371/journal.pone.0131866)
Supplement: S8 Fig — Enrichment blots of different gene sets either enriched in the dnMpl or control phenotype. Supplied are the normalized enrichment score (NES), the nominal p-value, and the false discovery rate (FDR). (PDF) [file pone.0131866.s008.pdf]

Kohlscheen, Wintterle et al., Supplementary Figure 8

Gene set up-regulated in HSC
(Jaatinen T et al., Stem Cells, 2006)

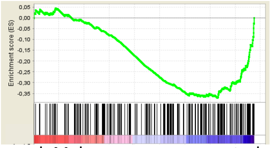

dnMpl control
NES= -1.68 p<0.001 FDR=0.01

Gene set down-regulated in HSC
(Jaatinen T et al., Stem Cells, 2006)

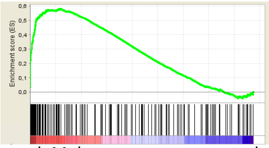

dnMpl control
NES=2.47 p<0.001 FDR<0.01

JAK -STAT -signaling pathway
(KEGG database)

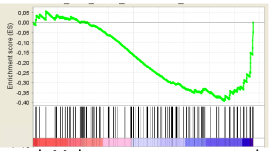

dnMpl control
NES= -1.59 p<0.05 FDR=0.02

LymphomyeloidDifferentiation
(GO database)

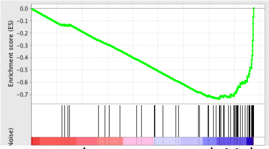

control dnMpl
NES= -2.70 p<0.001 FDR<0.01

Gene set in LT -HSC
(GazitR et al., J Exp Med, 2014)

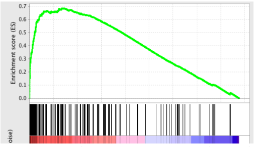

control dnMpl
NES=2.88 p<0.001 FDR<0.01

Mitosis
(GO database)

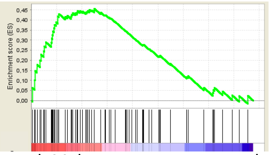

dnMpl control
NES=1.71 p<0.001 FDR=0.01
