# Supplementary material for: Inhibition of Thrombopoietin/Mpl Signaling in Adult Hematopoiesis Identifies New Candidates for Hematopoietic Stem Cell Maintenance
Source: PLoS One. 2015 Jul 6;10(7):e0131866. doi: 10.1371/journal.pone.0131866 (PMC4493002; doi:10.1371/journal.pone.0131866)
Supplement: S1 Table — The expression score is calculated as followed; (expression sample–expression control)/(SD sample + SD control), and the results ordered by the scores of the human genes. Expression scores lower -0.3 reflect the downregulation of the listed genes in both mouse and human. (PDF) [file pone.0131866.s012.pdf]

Supplementary Table 1: Gene list of murine and human gene expression score lower -0.3.

Gene symbol	Score M	Rank M	Score H	Rank H	Gene name
MMRN1	-0,47	21509	-1,72	17744	multimerin 1
ARHGAP6	-0,65	21792	-1,36	17740	Rho GTPase activating protein 6
MPL	-0,82	21865	-1,29	17738	myeloproliferative leukemia virus oncogene
TACSTD1	-0,32	20722	-1,25	17735	tumor-associated calcium signal transducer 1
RAB27B	-0,74	21848	-1,17	17727	RAB27B, member RAS oncogene family
SELP	-0,31	20669	-1,04	17711	selectin P (granule membrane protein 140kDa, antigen CD62)
RBPMS2	-0,57	21715	-1,03	17710	RNA binding protein with multiple splicing 2
TNFSF4	-0,35	20997	-1,01	17709	tumor necrosis factor (ligand) superfamily, member 4
NAP1L3	-0,78	21860	-0,97	17700	nucleosome assembly protein 1-like 3
CDC14B	-0,39	21211	-0,94	17696	CDC14 cell division cycle 14 homolog B (S. cerevisiae)
PDE5A	-0,58	21733	-0,90	17684	phosphodiesterase 5A, cGMP-specific
GUCY1A3	-0,65	21789	-0,86	17676	guanylate cyclase 1, soluble, alpha 3
TRPC6	-0,52	21655	-0,84	17668	transient receptor potential cation channel, subfamily C, member 6
MME	-0,30	20541	-0,74	17626	membrane metallo-endopeptidase (neutral endopeptidase, enkephalinase)
SDPR	-0,89	21877	-0,73	17617	serum deprivation response (phosphatidylserine binding protein)
CDC42BPA	-0,49	21569	-0,73	17615	CDC42 binding protein kinase alpha (DMPK-like)
MYCN	-0,49	21567	-0,70	17582	v-myc myelocytomatosis viral related oncogene, neuroblastoma derived (avian)
MYCT1	-0,43	21372	-0,69	17572	myc target 1
NUDT11	-0,31	20608	-0,64	17538	nudix (nucleoside diphosphate linked moiety X)-type motif 11
H1F0	-0,44	21419	-0,61	17512	H1 histone family, member 0
ARMCX1	-0,33	20787	-0,60	17495	armadillo repeat containing, X-linked 1
CRISPLD1	-0,45	21442	-0,60	17493	cysteine-rich secretory protein LCCL domain containing 1
MPP7	-0,70	21833	-0,59	17472	membrane protein, palmitoylated 7 (MAGUK p55 subfamily member 7)
MED12L	-0,33	20841	-0,58	17470	mediator of RNA polymerase II transcription, subunit 12 homolog (S. cerevisiae)-like
FHL1	-0,58	21727	-0,58	17451	four and a half LIM domains 1
BAALC	-0,31	20643	-0,57	17436	brain and acute leukemia, cytoplasmic
CDK2	-0,32	20757	-0,57	17435	cyclin-dependent kinase 2
CRISP3	-0,56	21707	-0,56	17433	cysteine-rich secretory protein 3
LY6G6D	-0,32	20741	-0,56	17422	lymphocyte antigen 6 complex, locus G6D
MPPED2	-0,42	21335	-0,54	17403	metallophosphoesterase domain containing 2
HGF	-0,30	20543	-0,52	17352	hepatocyte growth factor (hepapoietin A; scatter factor)
OSGEPL1	-0,49	21583	-0,51	17324	O-sialoglycoprotein endopeptidase-like 1
FNBP1L	-0,50	21592	-0,51	17319	formin binding protein 1-like
BNIP3	-0,53	21673	-0,50	17289	BCL2/adenovirus E1B 19kDa interacting protein 3
BTBD3	-0,36	21025	-0,50	17278	BTB (POZ) domain containing 3
HOXA10	-0,42	21337	-0,50	17275	homeobox A10
EREG	-0,42	21354	-0,48	17246	epiregulin
ME3	-0,36	21059	-0,47	17214	malic enzyme 3, NADP(+)-dependent, mitochondrial
SFXN4	-0,35	20929	-0,46	17188	sideroflexin 4
TBXAS1	-0,35	20927	-0,46	17176	thromboxane A synthase 1 (platelet, cytochrome P450, family 5, subfamily A)
SCCPDH	-0,47	21511	-0,45	17160	saccharopine dehydrogenase (putative)
ACRBP	-0,35	20952	-0,45	17144	acrosin binding protein
CACNA2D3	-0,50	21613	-0,43	17110	calcium channel, voltage-dependent, alpha 2/delta 3 subunit
NPAL3	-0,54	21689	-0,43	17098	NIPA-like domain containing 3
TCEAL1	-0,40	21247	-0,43	17093	transcription elongation factor A (SII)-like 1
ALS2CR4	-0,32	20731	-0,43	17088	amyotrophic lateral sclerosis 2 (juvenile) chromosome region, candidate 4
BCAT1	-0,41	21267	-0,42	17063	branched chain aminotransferase 1, cytosolic
RAB37	-0,60	21753	-0,42	17062	RAB37, member RAS oncogene family
HDGFRP3	-0,34	20887	-0,40	17034	hepatoma-derived growth factor, related protein 3
HOXA5	-0,30	20548	-0,40	17032	homeobox A5
NCKAP1	-0,43	21376	-0,40	17019	NCK-associated protein 1
BEX1	-0,47	21504	-0,40	17002	brain expressed, X-linked 1
DYNC2LI1	-0,53	21668	-0,39	16988	dynein, cytoplasmic 2, light intermediate chain 1
VWF	-0,39	21176	-0,39	16985	von Willebrand factor
KCTD15	-0,44	21438	-0,38	16931	potassium channel tetramerisation domain containing 15
XRCC4	-0,33	20770	-0,38	16922	X-ray repair complementing defective repair in Chinese hamster cells 4
SOCS2	-0,98	21885	-0,38	16914	suppressor of cytokine signaling 2
KCNK5	-0,88	21876	-0,37	16901	potassium channel, subfamily K, member 5
ANKRD50	-0,43	21391	-0,37	16896	ankyrin repeat domain 50
SPG3A	-0,31	20595	-0,37	16869	spastic paraplegia 3A (autosomal dominant)
ALDH6A1	-0,34	20874	-0,36	16854	aldehyde dehydrogenase 6 family, member A1
TRIP6	-0,47	21526	-0,36	16834	thyroid hormone receptor interactor 6
DSCR2	-0,33	20826	-0,35	16802	Down syndrome critical region gene 2
KDELC2	-0,42	21319	-0,35	16793	KDEL (Lys-Asp-Glu-Leu) containing 2
PFKM	-0,44	21414	-0,33	16691	phosphofructokinase, muscle
P2RX1	-0,43	21385	-0,33	16671	purinergic receptor P2X, ligand-gated ion channel, 1
GTPBP8	-0,33	20779	-0,32	16640	GTP-binding protein 8 (putative)
KCNMB1	-0,37	21121	-0,31	16558	potassium large conductance calcium-activated channel, subfamily M, beta member 1
LMNA	-0,37	21061	-0,30	16535	lamin A/C
ORC5L	-0,31	20570	-0,30	16524	origin recognition complex, subunit 5-like (yeast)
EHHADH	-0,38	21152	-0,30	16520	enoyl-Coenzyme A, hydratase/3-hydroxyacyl Coenzyme A dehydrogenase
