# Supplementary material for: Inhibition of Thrombopoietin/Mpl Signaling in Adult Hematopoiesis Identifies New Candidates for Hematopoietic Stem Cell Maintenance
Source: PLoS One. 2015 Jul 6;10(7):e0131866. doi: 10.1371/journal.pone.0131866 (PMC4493002; doi:10.1371/journal.pone.0131866)
Supplement: S2 Table — The expression score is calculated as followed; (expression sample–expression control)/(SD sample + SD control), and the results ordered by the scores of the human genes. Expression scores higher 0.3 reflect the upregulation of the listed genes in both mouse and human. (PDF) [file pone.0131866.s013.pdf]

Supplementary Table 2: Gene list of murine and human gene expression score higher 0.3.

Gene symbol	Score M	Rank M	Score H	Rank H	Gene name
OFD1	0,42	611	0,30	935	oral-facial-digital syndrome 1
EMR1	0,66	152	0,30	932	egf-like module containing, mucin-like, hormone receptor-like 1
QSCN6L1	0,50	385	0,31	895	quiescin Q6-like 1
MTUS1	1,08	21	0,32	849	mitochondrial tumor suppressor 1
MAK	0,40	707	0,32	848	male germ cell-associated kinase
KYNU	0,37	835	0,32	835	kynureninase (L-kynurenine hydrolase)
DOCK10	0,43	592	0,32	832	dedicator of cytokinesis 10
LRG1	0,49	399	0,32	831	leucine-rich alpha-2-glycoprotein 1
ITGAX	0,64	177	0,32	829	integrin, alpha X (complement component 3 receptor 4 subunit)
IGFBP5	0,59	225	0,32	827	insulin-like growth factor binding protein 5
DIRC2	0,43	593	0,32	826	disrupted in renal carcinoma 2
CCDC93	0,37	854	0,32	815	coiled-coil domain containing 93
STARD5	0,32	1153	0,33	793	START domain containing 5
SLC31A2	0,33	1106	0,33	771	solute carrier family 31 (copper transporters), member 2
KIF21B	0,33	1115	0,34	749	kinesin family member 21B
GPM6A	0,40	709	0,34	745	glycoprotein M6A
CD19	0,49	408	0,34	722	CD19 molecule
GRAMD3	0,33	1097	0,35	665	GRAM domain containing 3
ITFG1	0,35	935	0,37	630	integrin alpha FG-GAP repeat containing 1
RIN2	0,40	705	0,37	596	Ras and Rab interactor 2
NEDD9	0,57	254	0,38	581	neural precursor cell expressed, developmentally down-regulated 9
GZMB	0,35	968	0,39	521	granzyme B (granzyme 2, cytotoxic T-lymphocyte-associated serine esterase 1)
ICOSLG	0,43	573	0,39	513	inducible T-cell co-stimulator ligand
HMOX1	0,46	474	0,40	502	heme oxygenase (decycling) 1
ENC1	0,54	297	0,40	500	ectodermal-neural cortex (with BTB-like domain)
ACSL6	0,61	199	0,40	496	acyl-CoA synthetase long-chain family member 6
PDE4D	0,49	402	0,44	400	phosphodiesterase 4D, cAMP-specific (phosphodiesterase E3 dunce homolog, Drosophila)
PARP8	0,40	718	0,44	390	poly (ADP-ribose) polymerase family, member 8
CAMSAP1L1	0,38	786	0,45	365	calmodulin regulated spectrin-associated protein 1-like 1
TMEM2	0,39	733	0,45	364	transmembrane protein 2
C1QC	0,53	321	0,46	349	complement component 1, q subcomponent, C chain
KBTBD8	0,61	195	0,48	297	kelch repeat and BTB (POZ) domain containing 8
SYNE1	0,37	857	0,48	284	spectrin repeat containing, nuclear envelope 1
CAV1	0,36	912	0,49	267	caveolin 1, caveolae protein, 22kDa
IGSF6	0,43	566	0,50	254	immunoglobulin superfamily, member 6
RGL1	0,30	1286	0,50	252	ral guanine nucleotide dissociation stimulator-like 1
KLRC3	0,33	1091	0,51	249	killer cell lectin-like receptor subfamily C, member 3
ST3GAL5	0,47	437	0,51	239	ST3 beta-galactoside alpha-2,3-sialyltransferase 5
EGR2	0,68	133	0,51	238	early growth response 2 (Krox-20 homolog, Drosophila)
FAIM3	0,39	762	0,52	225	Fas apoptotic inhibitory molecule 3
ALOX5	0,55	289	0,53	205	arachidonate 5-lipoxygenase
ADAM19	0,50	382	0,53	204	ADAM metallopeptidase domain 19 (meltrin beta)
KLRD1	0,48	411	0,53	202	killer cell lectin-like receptor subfamily D, member 1
IL18RAP	0,32	1181	0,55	188	interleukin 18 receptor accessory protein
CNN3	0,47	438	0,57	172	calponin 3, acidic
C1QA	0,54	308	0,58	164	complement component 1, q subcomponent, A chain
FCGR2B	0,72	112	0,59	143	Fc fragment of IgG, low affinity IIb, receptor (CD32)
TULP4	0,52	350	0,60	141	tubby like protein 4
PLEKHA1	0,32	1143	0,61	124	pleckstrin homology domain containing, family A (phosphoinositide binding specific) member 1
SIPA1L2	0,39	749	0,62	110	signal-induced proliferation-associated 1 like 2
PLA2G7	0,48	409	0,64	95	phospholipase A2, group VII (platelet-activating factor acetylhydrolase, plasma)
CD5L	0,37	830	0,64	94	CD5 molecule-like
DUSP4	0,38	797	0,68	76	dual specificity phosphatase 4
ICOS	0,45	499	0,70	67	inducible T-cell co-stimulator
CD3D	0,37	864	0,70	62	CD3d molecule, delta (CD3-TCR complex)
FCRL1	0,34	1000	0,72	59	Fc receptor-like 1
CD80	0,39	726	0,76	47	CD80 molecule
CD8A	0,31	1231	0,77	45	CD8a molecule
ABCB4	0,44	540	0,79	38	ATP-binding cassette, sub-family B (MDR/TAP), member 4
RASGRP1	0,42	625	0,81	33	RAS guanyl releasing protein 1 (calcium and DAG-regulated)
C1QB	0,42	616	0,83	26	complement component 1, q subcomponent, B chain
SLAMF7	0,60	214	0,86	22	SLAM family member 7
MS4A1	0,91	50	0,87	19	membrane-spanning 4-domains, subfamily A, member 1
KMO	0,53	319	0,90	18	kynurenine 3-monooxygenase (kynurenine 3-hydroxylase)
GZMA	0,45	496	0,95	12	granzyme A (granzyme 1, cytotoxic T-lymphocyte-associated serine esterase 3)
CDK5R1	0,32	1184	1,27	2	cyclin-dependent kinase 5, regulatory subunit 1 (p35)
