# Supplementary material for: Inhibition of Thrombopoietin/Mpl Signaling in Adult Hematopoiesis Identifies New Candidates for Hematopoietic Stem Cell Maintenance
Source: PLoS One. 2015 Jul 6;10(7):e0131866. doi: 10.1371/journal.pone.0131866 (PMC4493002; doi:10.1371/journal.pone.0131866)
Supplement: S3 Table — The expression score is calculated as followed; (expression sample–expression control)/(SD sample + SD control), and the results ordered by the scores of the human genes. Expression scores higher 0.3 in human and lower -0.3 in mouse reflect the upregulation and downregulation of the listed genes, respectively. (PDF) [file pone.0131866.s014.pdf]

Supplementary Table 3: Gene list of murine gene expression score higher 0.3 and human gene expression score lower -0.3.

Gene symbol	Score M	Rank M	Score H	Rank H	Gene name
SLC35D3	0,41	663	-1,69	17743	solute carrier family 35, member D3
ELA2	0,72	116	-1,00	17705	elastase 2, neutrophil
PROM1	0,93	45	-0,97	17702	prominin 1
PRG2	0,61	201	-0,91	17691	proteoglycan 2, bone marrow (natural killer cell activator, eosinophil granule major basic protein)
FCER1A	0,75	90	-0,91	17689	Fc fragment of IgE, high affinity I, receptor for; alpha polypeptide
ESCO2	0,38	792	-0,88	17683	establishment of cohesion 1 homolog 2 (S. cerevisiae)
KIF15	0,51	375	-0,87	17680	kinesin family member 15
B4GALT6	0,30	1282	-0,86	17678	UDP-Gal:betaGlcNAc beta 1,4- galactosyltransferase, polypeptide 6
CTSG	0,68	137	-0,84	17667	cathepsin G
SERPINE2	0,42	608	-0,84	17666	serpin peptidase inhibitor, clade E (nexin, plasminogen activator inhibitor type 1), member 2
SSX2IP	0,45	494	-0,82	17659	synovial sarcoma, X breakpoint 2 interacting protein
RAG1	0,65	164	-0,81	17652	recombination activating gene 1
DLG7	0,31	1255	-0,79	17649	discs, large homolog 7 (Drosophila)
RHAG	1,46	2	-0,78	17643	Rh-associated glycoprotein
MS4A3	0,93	44	-0,72	17607	membrane-spanning 4-domains, subfamily A, member 3 (hematopoietic cell-specific)
RAG2	0,47	429	-0,71	17596	recombination activating gene 2
EPX	0,56	261	-0,69	17579	eosinophil peroxidase
AURKA	0,43	580	-0,68	17568	aurora kinase A
KIF18A	0,53	323	-0,65	17544	kinesin family member 18A
GPSM2	0,38	764	-0,63	17526	G-protein signalling modulator 2 (AGS3-like, C. elegans)
TMEM97	0,31	1229	-0,61	17510	transmembrane protein 97
TTC7B	0,32	1169	-0,58	17469	tetratricopeptide repeat domain 7B
F13A1	0,47	453	-0,55	17414	coagulation factor XIII, A1 polypeptide
PRC1	0,46	479	-0,54	17396	protein regulator of cytokinesis 1
KIF2C	0,36	871	-0,54	17388	kinesin family member 2C
MAD2L1	0,31	1219	-0,52	17357	MAD2 mitotic arrest deficient-like 1 (yeast)
PARVB	0,32	1168	-0,52	17334	parvin, beta
MELK	0,74	101	-0,52	17331	maternal embryonic leucine zipper kinase
SLC10A4	0,65	169	-0,51	17313	solute carrier family 10 (sodium/bile acid cotransporter family), member 4
COG6	0,33	1066	-0,49	17272	component of oligomeric golgi complex 6
RHD	0,44	560	-0,49	17257	Rh blood group, D antigen
ANXA1	0,37	818	-0,49	17252	annexin A1
NUCB2	0,71	122	-0,48	17227	nucleobindin 2
FHIT	0,99	30	-0,47	17222	fragile histidine triad gene
SUHW4	0,32	1146	-0,46	17182	suppressor of hairy wing homolog 4 (Drosophila)
HDC	0,48	427	-0,45	17162	histidine decarboxylase
MCM2	0,34	997	-0,45	17147	MCM2 minichromosome maintenance deficient 2, mitotin (S. cerevisiae)
DNA2L	0,44	537	-0,44	17139	DNA2 DNA replication helicase 2-like (yeast)
SERAC1	0,35	939	-0,44	17135	serine active site containing 1
P2RY12	0,47	431	-0,43	17103	purinergic receptor P2Y, G-protein coupled, 12
KLF1	0,50	380	-0,43	17090	Kruppel-like factor 1 (erythroid)
SLC44A1	0,38	765	-0,42	17077	solute carrier family 44, member 1
CAMP	0,67	144	-0,41	17049	cathelicidin antimicrobial peptide
CLEC5A	0,89	52	-0,39	16964	C-type lectin domain family 5, member A
RCC1	0,51	358	-0,38	16936	regulator of chromosome condensation 1
E2F2	0,68	139	-0,38	16923	E2F transcription factor 2
EIF2B3	0,48	415	-0,38	16908	eukaryotic translation initiation factor 2B, subunit 3 gamma, 58kDa
CA1	0,95	38	-0,37	16884	carbonic anhydrase I
RARSL	0,35	979	-0,37	16873	arginyl-tRNA synthetase-like
MGST2	0,63	183	-0,37	16868	microsomal glutathione S-transferase 2
LRBA	0,32	1187	-0,37	16863	LPS-responsive vesicle trafficking, beach and anchor containing
CEP76	0,39	727	-0,37	16859	centrosomal protein 76kDa
PIK3CB	0,41	670	-0,36	16835	phosphoinositide-3-kinase, catalytic, beta polypeptide
PSTPIP2	0,63	181	-0,35	16774	proline-serine-threonine phosphatase interacting protein 2
PLK1	0,35	956	-0,34	16746	polo-like kinase 1 (Drosophila)
SNCA	0,44	552	-0,34	16718	synuclein, alpha (non A4 component of amyloid precursor)
GTF2H5	0,38	773	-0,33	16696	general transcription factor IIH, polypeptide 5
LATS2	0,39	737	-0,33	16681	LATS, large tumor suppressor, homolog 2 (Drosophila)
ENDOD1	0,43	601	-0,32	16651	endonuclease domain containing 1
NET1	0,45	510	-0,32	16646	neuroepithelial cell transforming gene 1
UGDH	0,31	1210	-0,32	16619	UDP-glucose dehydrogenase
ACTN1	0,46	465	-0,31	16592	actinin, alpha 1
STEAP3	0,40	706	-0,31	16566	STEAP family member 3
ADAL	0,33	1072	-0,30	16540	adenosine deaminase-like
ATP8B4	0,51	353	-0,30	16536	ATPase, Class I, type 8B, member 4
