# Supplementary material for: Inhibition of Thrombopoietin/Mpl Signaling in Adult Hematopoiesis Identifies New Candidates for Hematopoietic Stem Cell Maintenance
Source: PLoS One. 2015 Jul 6;10(7):e0131866. doi: 10.1371/journal.pone.0131866 (PMC4493002; doi:10.1371/journal.pone.0131866)
Supplement: S4 Table — The expression score is calculated as followed; (expression sample–expression control)/(SD sample + SD control), and the results ordered by the scores of the human genes. Expression scores higher 0.3 in mouse and lower -0.3 in human reflect the upregulation and downregulation of the listed genes, respectively. (PDF) [file pone.0131866.s015.pdf]

Supplementary Table 4: Gene list of murine gene expression score lower -0.3 and human expression score higher 0.3.

Gene symbol	Score M	Rank M	Score H	Rank H	Gene name
ASB1	-0,42	21357	0,30	940	ankyrin repeat and SOCS box-containing 1
ADAMTS9	-0,31	20676	0,30	936	ADAM metallopeptidase with thrombospondin type 1 motif, 9
PODXL	-0,41	21294	0,30	928	podocalyxin-like
UBASH3A	-0,40	21236	0,31	921	ubiquitin associated and SH3 domain containing, A
MORC4	-0,63	21778	0,31	918	MORC family CW-type zinc finger 4
RAB22A	-0,33	20775	0,31	890	RAB22A, member RAS oncogene family
DBNDD2	-0,44	21407	0,31	884	dysbindin (dystrobrevin binding protein 1) domain containing 2
MX2	-0,40	21263	0,31	881	myxovirus (influenza virus) resistance 2 (mouse)
CHRM3	-0,33	20810	0,31	868	cholinergic receptor, muscarinic 3
NEB	-0,37	21104	0,33	807	nebulin
SELM	-0,38	21136	0,34	759	selenoprotein M
TTC9	-0,33	20799	0,34	753	tetratricopeptide repeat domain 9
SDC2	-0,33	20846	0,35	711	syndecan 2 (heparan sulfate proteoglycan 1, cell surface-associated, fibroglycan)
COL4A2	-0,46	21480	0,35	697	collagen, type IV, alpha 2
KIF3A	-0,36	21056	0,35	675	kinesin family member 3A
DIP2C	-0,64	21786	0,37	619	DIP2 disco-interacting protein 2 homolog C (Drosophila)
SLFN5	-0,33	20836	0,38	583	schlafen family member 5
GIMAP1	-0,47	21503	0,38	572	GTPase, IMAP family member 1
FOXC2	-0,38	21148	0,38	556	forkhead box C2 (MFH-1, mesenchyme forkhead 1)
GHR	-0,40	21255	0,40	492	growth hormone receptor
WIF1	-0,32	20715	0,42	430	WNT inhibitory factor 1
IL12RB1	-0,52	21640	0,42	428	interleukin 12 receptor, beta 1
SCML4	-0,51	21623	0,43	405	sex comb on midleg-like 4 (Drosophila)
F3	-0,35	20943	0,44	396	coagulation factor III (thromboplastin, tissue factor)
CCL4	-0,42	21325	0,44	382	chemokine (C-C motif) ligand 4
TJP1	-0,45	21463	0,44	381	tight junction protein 1 (zona occludens 1)
TLR4	-0,61	21763	0,45	360	toll-like receptor 4
E2F5	-0,69	21822	0,46	341	E2F transcription factor 5, p130-binding
MAML2	-0,41	21297	0,46	331	mastermind-like 2 (Drosophila)
CRTAM	-0,31	20657	0,47	308	cytotoxic and regulatory T cell molecule
DIP2A	-0,38	21144	0,48	300	DIP2 disco-interacting protein 2 homolog A (Drosophila)
HRASLS3	-0,36	21001	0,49	274	HRAS-like suppressor 3
DENND1C	-0,43	21382	0,50	258	DENN/MADD domain containing 1C
GBP2	-0,35	20955	0,50	250	guanylate binding protein 2, interferon-inducible
RGS1	-0,38	21164	0,52	232	regulator of G-protein signalling 1
GIMAP8	-0,66	21803	0,52	228	GTPase, IMAP family member 8
FBXO32	-0,41	21305	0,53	212	F-box protein 32
GPRASP1	-0,53	21671	0,53	207	G protein-coupled receptor associated sorting protein 1
NR4A2	-0,49	21568	0,54	193	nuclear receptor subfamily 4, group A, member 2
BAG3	-0,43	21398	0,57	170	BCL2-associated athanogene 3
TGFBR3	-0,35	20951	0,59	153	transforming growth factor, beta receptor III (betaglycan, 300kDa)
RASGEF1B	-0,46	21467	0,59	149	RasGEF domain family, member 1B
MATN1	-0,30	20547	0,62	111	matrilin 1, cartilage matrix protein
SASH1	-0,67	21805	0,67	77	SAM and SH3 domain containing 1
DMD	-0,43	21366	0,69	72	dystrophin (muscular dystrophy, Duchenne and Becker types)
IL15	-0,69	21829	0,70	66	interleukin 15
LCK	-0,49	21565	0,74	57	lymphocyte-specific protein tyrosine kinase
GATA3	-0,37	21066	0,76	46	GATA binding protein 3
SPP1	-0,47	21508	0,79	36	secreted phosphoprotein 1 (osteopontin, bone sialoprotein I, early T-lymphocyte activation 1)
RRAS2	-0,37	21069	0,86	23	related RAS viral (r-ras) oncogene homolog 2
SLAMF1	-0,32	20690	0,96	11	signaling lymphocytic activation molecule family member 1
